# Whole-genomic and transcriptomic analyses elucidate *p*-cresol and styrene degradation metabolism in *Rhodococcus opacus* 1CP

**DOI:** 10.1128/aem.00045-26

**Published:** 2026-03-27

**Authors:** Selvapravin Kumaran, Thomas Heine, Janosch A. D. Gröning, Michael Schlömann, Andreas Albersmeier, Tobias Busche, Jörn Kalinowski, Christian Rückert-Reed, Lena Schaffert, Dirk Tischler

**Affiliations:** 1 Microbial Biotechnology, Faculty for Biology and Biotechnology, Ruhr University Bochum 9142, Bochum, Germany; 2 Faculty of Chemistry, Physics and Biosciences, Institute of Biosciences, TU Bergakademie Freiberg 26545, Freiberg, Germany; 3 Technology Platform Genomics, Center for Biotechnology (CeBiTec), Bielefeld University 9167, Bielefeld, Germany; 4 Medical School OWL, Bielefeld University 9167, Bielefeld, Germany; Shanghai Jiao Tong University, Shanghai, China

**Keywords:** *Rhodococcus opacus *1CP, phenol hydroxylases, *ortho*-/*meta*-/side chain attack, *pheA1 *knock-out, transcriptome, gene redundancy, lignin metabolization

## Abstract

The genus *Rhodococcus* comprises numerous strains recognized for degradation of pollutants and production of secondary metabolites, including biosurfactants, lignin breakdown, and utilization of volatile organic compounds. Often, gene redundancies and the evolution of alternative pathways are attributed to such characteristics. *Rhodococcus opacus* 1CP, initially isolated as a chlorophenol-degrading strain, was found to be a model organism comprising several such features. In this study, we analyzed the genome and transcriptome and provided evidence that the strain 1CP uses three different pathways of *ortho-*, *meta-*, and side-chain attack for the degradation of aromatic compounds. The wild-type strain and the single and double knock-out mutants of phenol hydroxylases were able to attack substituted phenols via the classical *ortho*-route. In contrast, the triple knock-out mutant expressed *meta*-pathway genes to act on *p*-cresol, indicating that this pathway serves as a reserve in strain 1CP. Growth of 1CP on phenol, *p*-cresol, and styrene induces several gene clusters that are associated with lignin metabolization. Catechol, protocatechuate, and phenylacetic acid are major key intermediates that are funneled into central metabolic pathways, which enable strain 1CP to degrade acetophenone, benzoate, phenol, 2-phenylethanol, and styrene. Strain 1CP possesses an alternative, modified *ortho*-cleavage pathway that allows it to degrade 2-chlorophenol. Interestingly, in almost all cases, redundant genes were identified; however, only in a few cases, such as phenol hydroxylases, were they found to be active and simultaneously involved in metabolic activities. The transcriptomic and kinetic data showed that the redundant styrene oxide isomerase is upregulated and involved in styrene degradation.

**IMPORTANCE:**

*Rhodococcus opacus* 1CP is a model organism for various biotechnological applications due to its capabilities to metabolize a vast range of aromatic and xenobiotic compounds. Although strain 1CP has been used for decades in bioremediation, the complete metabolic pathways underlying degradation have never been elucidated. In this study, the ability of the strain to bypass phenol hydroxylase deletions and degrade substituted phenols is described using genomics, transcriptomics, and gene knock-out analyses. Despite its metabolic versatility, strain 1CP has been reported only for the *ortho*-cleavage pathway. No enzymatic or metabolic evidence has supported the presence of a *meta*-cleavage pathway to degrade aromatic compounds. Genes associated with such *meta*-pathways have been identified, but are not clustered with known degradation operons. In this study, we demonstrate that a triple knock-out mutant can utilize a *meta*-cleavage pathway for the degradation of substituted phenols.

## INTRODUCTION

The genus *Rhodococcus* has been extensively reported to produce antibiotics and other important natural products. Recently, they were found to act on isoprene compounds (1–5) and are therefore important biocatalytic tools in biotechnological applications (6–9). G+C- rich, aerobic, mycolate-containing actinomycetes, *Rhodococcus* strains have been isolated from various environments (10, 11). For instance, the strain *Rhodococcus baikonurensis* was isolated from the Russian space station *Mir* (12). *Rhodococcus* strains are of interest because of their ability to degrade a broad range of xenobiotic compounds, including (halo-)aromatic compounds, polycyclic aromatic compounds, heteroaromatic nitriles, and long-chain *n*-alkanes (11, 13–17, 18). In addition, they have been reported to degrade complex compounds, such as plastics (polyethylene and polystyrene), plasticizers (*n*-butyl benzyl phthalate), lignin, rubber, and steroids (19–24). Their metabolic versatility is mainly due to the large variety of enzymes encoded in circular or linear plasmids and large genomes (7–9 Mbp) (25–27).

The representative strain *Rhodococcus erythropolis* 1CP was isolated as a chlorophenol degrader (28, 29) and later reclassified as *R. opacus* (30) in 1999. Since then, the strain has been subjected to various investigations regarding the degradation and production of various compounds, stress response, and biotechnological applications (Table S1) (31–35). Several metabolic pathways and their key enzymes have been characterized, including dye degradation (36); formation of maleimides, pigments, epoxides, or sulfoxides (37–40); and biosurfactant production (41, 42).

Strain 1CP was studied intensively for (halo-)aromatic compound degradation, which includes the degradation pathways for styrene, benzoate, phenol, and catechol derivatives(43–45) . In particular, the degradation of 4-chlorophenol and 2,4-dichlorophenol has been investigated (28). Degradation of 3-chlorophenol is less efficient, while 2-chlorophenol can only be transformed after prolonged adaptation (46). As an adaptive mechanism, strain 1CP is also able to change its lipid composition within the membrane depending on the growth medium. This results in a 10-fold increase in methyl-branched fatty acids when phenol or 4-chlorophenol is present as substrate, compared to fructose (30).

Phenols (or substituted phenols) and benzoates are activated by various mono- and dioxygenases (Table 1). Some of them, such as phenol hydroxylases, are encoded in several copies on the 1CP genome (47). Depending on the substituents, the activated products are further funneled into protocatechuate and (halo-)catechol degradative pathways. These different sets of gene clusters have been subjected to various studies (48–50). In particular, the degradation of 3-chlorocatechol appeared to be interesting, as it follows a modified *ortho*-cleavage pathway (48, 51).

**TABLE 1:** All genes/gene clusters of 1CP applied for biocatalysis in 1CP^*a*^

Biocatalysts	Accession no.^*b*^	Location	Reference
Benzoate 1,2-dioxygenase (BenABC)	WP_065492188/ WP_005258735/ WP_065492189 (**ξ**)	{C70}	(52)
3-Chlorobenzoate 1,2-dioxygenase			(53, 54)
3-Hydroxybenzoate 6-hydroxylase	WP_065491985	C	(55)
2,5-Dihydroxybenzoate dioxygenase	WP_065491982	C	(55)
Protocatechuate 3,4-dioxygenase (PcaHG)	WP_065491756/WP_005261317 (**$**)	{C57}	(50)
3-Carboxymuconate cycloisomerase (PcaB)	WP_065491757 (**$**)	{C57}	
4-Carboxymuconolactone decarboxylase/ 3-oxoadipate enollactone hydrolase (PcaL)	WP_065491758 (**$**)	{C57}	
(Chloro)phenol hydroxylases (PheA1/PheA2)	WP_032492681/WP_032492682 (*)	{I104}	(47), this study
	WP_005264050/WP_037231006 (**ξ**)	{C68}	
	WP_065492265/ WP_065492264 (**‡**)	{C75}	
Catechol 1,2-dioxygenase (CatA, CatA2, CatA3, CatA4)	WP_065492183 (**ξ**)	{C65}	(47, 49, 53, 56), this study
	WP_065492266 (**‡**)	{C75}	
	WP_005264055 (**ξ**)	{C67}	
	WP_065493784 (*)	{I105}	
Muconate cycloisomerase (CatB)	WP_065492182 (**ξ**)	{C65}	(50, 57)
Muconolactone isomerase (CatC)	WP_005573881 (**ξ**)	{C65}	(49)
IclR-type regulator (CatR)	WP_005264056 (**ξ**)	{C65}	(58)
4-Chlorocatechol 1,2-dioxygenase (ClcA)^α^	WP_065493674 (**χ**)	{I97}	(59–62)
Chloromuconate cycloisomerase (ClcB)^α^	WP_081315590 (**χ**)	{I97}	(57)
Dienelactone hydrolase (ClcD)^α^	WP_065493673 (**χ**)	{I97}	(63)
3-Chlorocatechol 1,2-dioxygenase (ClcA2)	WP_032492675 (*)	{I103}	(48, 51, 62, 64, 65)
Chloromuconate cycloisomerase (ClcB2)	WP_065493777 (*)	{I103}	(57, 66)
5-Chloromuconolactone dehalogenase (ClcF)	WP_032492678 (*)	{I103}	(65, 67–71)
Dienelactone hydrolase (ClcD2)	WP_032492676WP_032492676 (*)	{I103}	(63)
Maleylacetate reductase (ClcE, ClcE2, MacA)	WP_065493910 (*)	{I104}	
	WP_081315596 ^β^	I	(72)
Succinyl-CoA:3-oxoadipate CoA transferase (PcaIJ)	WP_005248111/WP_005261315 (**$**)	{C57}	(50)
3-Oxoadipyl CoA thiolase (PcaF)	WP_065491759 (**$**)	{C57}	(50)
Styrene monooxygenase (StyA/B)	WP_065493737/WP_065493736 (**Ф**)	{I102}	(73)
Styrene oxide isomerase (StyC)	WP_065493735 (**Ф**)	{I102}	(74, 75)
Indole monooxygenases (IndA1 & IndA2B)	WP_065490891/WP_065490893	{C46}	(76–78)
Trehalose phosphate synthases (OstA1 & OstA2)	WP_065491169/WP_065489169	C	(42)
Ene-reductase (OYERo2)	WP_064080187	C	(37)
Azo-reductases (AzoRo)	WP_065493914	I	(36, 79, 80)
UDP-glucose pyrophosphorylases (*Ro*GalU1 & *Ro*GalU2)	WP_065489819/WP_005567389	C	(81)

^*a*^ Location of the genes is indicated by (C) chromosome or (Ι) pR1CP1. When applicable, the gene(s) is matched with the corresponding number in Data Set S1 (shown in parentheses in the location column). PheA2(1) was not expressed successfully. Genes assigned with Greek letters are located in repeat regions: α, 22^505573-517760^ and 23^517823-616758^; β, 23 and 24^616821-704469^.

^*b*^ Symbols indicate genes that belong to the same functional cluster. Genes associated with the *ortho*-cleavage pathway are marked with (**ξ**), while those for the *ß*-ketoadipate pathway are marked with (**$**). The chlorocatechol pathway is marked with (**χ**), (**‡**), and (*), and the styrene degradation pathway is marked with (**Ф**).

Herein, the cycloisomerase ClcB2 is unable to dehalogenate the 5-chloromuconolactone during the transformation of 2-chloro-*cis*,*cis*-muconate. Thus, an additional dehalogenase (ClcF), related to the muconolactone isomerase family, is required for the formation of dienelactone. It is the only representative of this type of dehalogenases identified so far (48, 67). The dienelactone is transformed to maleylacetate by dienelactone hydrolases and converted to 3-oxoadipate by maleylacetate reductase (ClcE or MacA), the central intermediate of this degradation pathway in strain 1CP (63). These reductases appear to be encoded several times on the genome, and at least one of them could perform the dehalogenation of chloromaleylacetate during the degradation of di- and trihalogenated catechols (72).

Although strain 1CP encodes *meta*-route degradative genes, only (adapted) *ortho*-routes are utilized, which is also the case for *p-*cresol degradation. After activation by phenol hydroxylases, the methylcatechol is cleaved by an unspecific catechol 1,2-dioxygenase, followed by a specialized 3-methylmuconate cycloisomerase (82). However, further steps remained speculative.

Based on that previous work (82), a triple deletion mutant (1CP^Δ123^) was generated in this study, where the three phenol hydroxylases, which are associated with the *ortho*-degradative gene clusters (Table 1) (47), were knocked out from the wild-type strain (1CP^WT^). In addition, the initial activation of styrene in strain 1CP has not been resolved yet. Strain 1CP was the first representative, where two group E monooxygenase systems were identified in one strain and characterized (76). One of these monooxygenases (styrene monooxygenase – SMO; StyAB) is embedded in a styrene degradation gene cluster known from other bacteria. While both of the group E monooxygenases can epoxidize styrene, it remained unclear if the group E monooxygenase, which is not located in the styrene degradation cluster (initially designated StyA1 and StyA2B), is also involved in styrene metabolism (83). A more recent study demonstrated that the latter system is involved in the degradation of the styrene analog indole and, hence, has been classified as indole monooxygenases (IMOs) (84). In addition, 1CP contains two styrene oxide isomerases (*styC1* and *styC2*), with *styC1* being located in the styrene degradation gene cluster next to SMO, while the second copy is located 8,450 bp downstream. The former has been studied for isomerase activity (85, 86), while the function of the latter remains unsolved.

In this study, we report the complete closed genome of strain 1CP and conduct a whole-transcriptome analysis of phenol^WT^-, styrene^WT^-, tetradecane^WT^-, and *p-*cresol^Δ123^-grown cells to gain insights into the actual utilization of the enzymatic toolset for their specific degradation pathways. Analysis of the triple mutant 1CP^Δ123^ on *p-*cresol showed that this strain can bypass the blocked pathways and employs a so-far unused *meta*-degradation pathway. In addition, we also expressed and purified both styrene oxide isomerases heterologously in *E. coli*, and their activities are discussed.

## RESULTS

### *Rhodococcus opacus* 1CP: growth, morphology, and chemotaxis studies

Colonies grown on minimal medium (MM) agar plates after 72 h of incubation with benzoate as the carbon source showed a yellow, convex, circular, creamy appearance, with diameters ranging from 1 to 2 mm (Fig. S1). Log-phase cells appeared as curved rods under light microscopy (Fig. S2). Electron microscopy showed that the cells were branched and formed filamentous structures (Fig. S3). In addition to previously reported carbon sources, strain 1CP was able to grow on a wide variety of amino acids, mono- and disaccharides, alkanes, (aromatic) carboxylic acids, aromatic hydrocarbons, alditols, steroids, and complex media, which are summarized in Table S2.

### Genome assembly, annotation, and analysis

The genome of strain 1CP was determined to be 8,637,535 bp, consisting of a circular chromosome and two linear plasmids (pR1CP1 and pR1CP2), with an average G+C content of 67%. The complete genome statistics are given in Table S3. Over 73% of the genes were assigned to the clusters of orthologous genes (COG) categories (Table S4), of which 24% were involved in transport and metabolism of lipids, amino acids, or carbohydrates (COG categories E, G, and I in Table S4), implicating the catabolic versatility of strain 1CP. At least 6% of the genes seem to be involved in biosynthesis, transport, and catabolism of secondary metabolites (COG category Q in Table S4). Only 23% of the chromosome was annotated with a biological function by the RAST platform (Table S5). Thus, a low success rate in annotation indicated the necessity of manual curation in screening relevant gene clusters. In total, 107 genes/gene clusters involved in aromatic compound degradation were identified across the chromosome and plasmids. The complete list is provided in Data Set S1, with identifiers (IDs) indicated in curly brackets.

**Fig 1:**
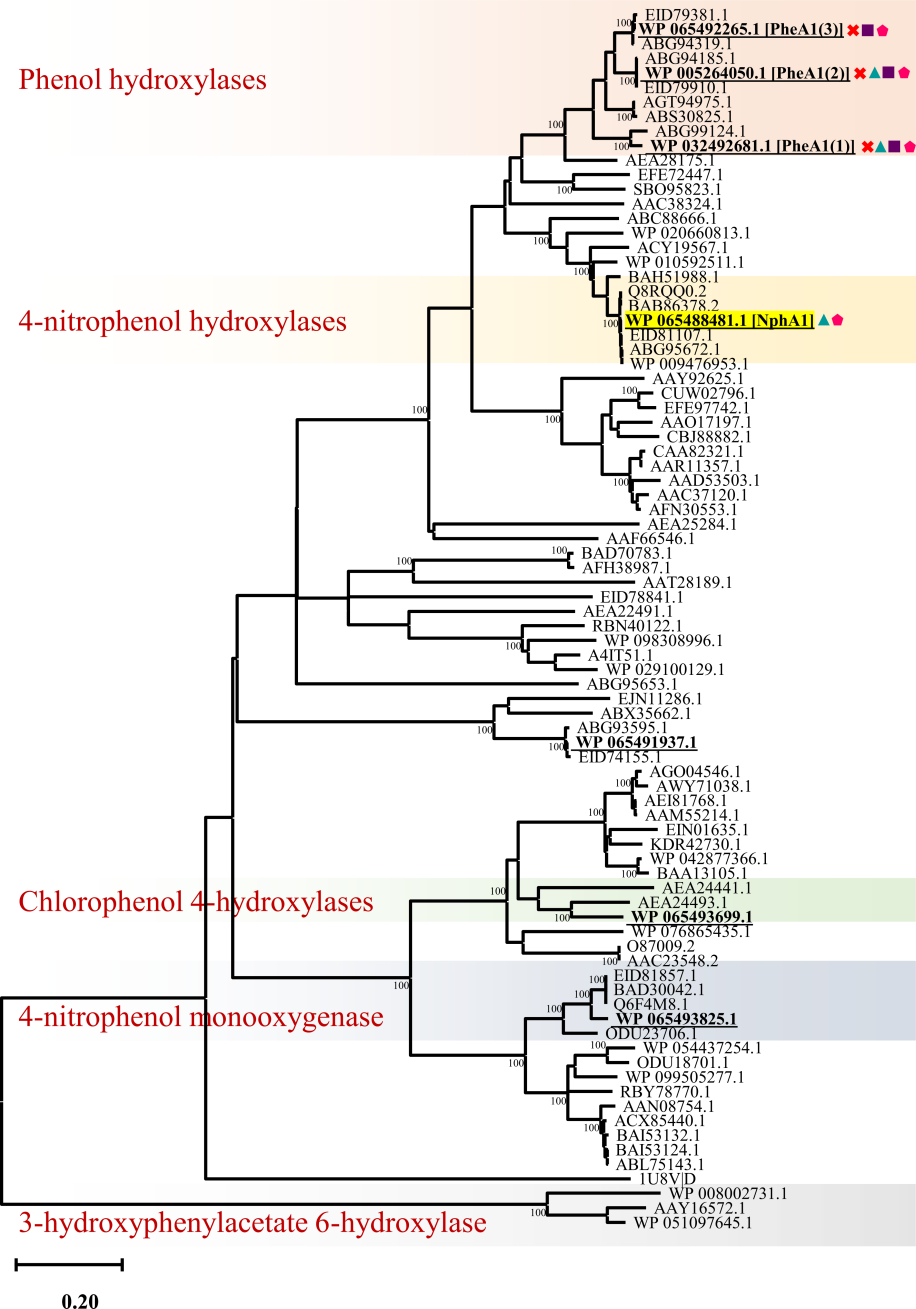
Phylogenetic tree of group D flavoprotein monooxygenases containing phenol hydroxylases and homologous proteins. *Rhodococcus opacus* 1CP owns seven representatives (indicated as bold and underlined), of which three [PheA1 (1–3)] were investigated in previous studies (47). These three phenol hydroxylases were knocked out in the triple mutant 1CP^Δ123^ (indicated by red crosses). A positive response of group D monooxygenases from strain 1CP on transcriptome level toward a respective substrate is highlighted by colored shapes (green triangle, Styrene^WT^; violet square, phenol^WT^; pink pentagon, *p*-cresol^Δ123^). The three phenol hydroxylases are expressed in the wild-type strain when phenol is added as a carbon source. The *p*-nitrophenol monooxygenase, which is supposed to overcome deletions in 1CP^Δ123^, is marked in yellow. Phenol hydroxylases are, to date, not known to hydroxylate styrene, which suggests that styrene only acts as an inducer due to structural similarity toward phenol. The expression of *pheA1 (1–3*) is induced in 1CP^Δ123^ but does not result in the formation of active protein, as essential parts are deleted.

Strain 1CP was reported to possess multiple redundant gene clusters for aromatic compound degradation. The initial activation of aromatic compounds is accomplished by mono- or dioxygenases. Strain 1CP has been reported to possess three active phenol hydroxylases (38). However, *in silico* analysis of the whole genome revealed four additional group D flavin monooxygenases, which might act on phenol derivatives (Fig. 1). Furthermore, several other monooxygenases are encoded, including group A flavin-dependent monooxygenases (3-hydroxycinnamic acid hydroxylase {C13}, pentachlorophenol 4-monooxygenase {C24}, 3-hydroxybenzoate 6-hydroxylase {C59}, 4-hydroxybenzoate 3-hydroxylase {C77}), Baeyer-Villiger monooxygenases {C14, C39, C53, C73, C86, C89}, and cytochrome P450-monooxygenases {C18, C19, C28, C64}. In addition, aromatic-ring-hydroxylating dioxygenases {C79, C90, C91, I95}, benzoate dioxygenase {C70}, and genes involved in converting benzoate to catechol were also identified.

Although strain 1CP has been reported to perform only *ortho-*cleavage of dehydroxylated intermediates mediated by catechol dioxygenases, the genome showed the presence of gene cluster *catRABC* {C65} on the chromosome and a *catA* homolog (*catA2*) on pR1CP1, located next to the phenol hydroxylase genes *pheA1*(3) {C75} and *pheA1*(1) {I104}, respectively. Even though *meta-*degradation has not been reported for 1CP, genes encoding catechol 2,3-dioxygenases {C2, C3} and other extradiol dioxygenases {C16, C54, C73} were identified in the genome. Furthermore, genes for aromatic demethylases {C69, I99}, which might act on methoxylated catecholic intermediates, were also observed.

Due to the differences in G+C content of the plasmids to the chromosome (Table S3), a horizontal gene transfer analysis was performed against the reference genome of *Rhodococcus opacus* B4. This indicated that both plasmids might be of alien origin (Fig. S4, 87). The megaplasmid pR1CP1 (formerly designated as p1CP) contains a large number of catabolic genes, such as degradation gene clusters for chlorocatechols (*clcDARB* {I97} and *clcA2D2B2F* {I103}) and styrene (*styABCD* {I102}). It also contains several repeat regions comprising an identical copy of the 4-chlorocatechol degradation gene cluster (*clcDARB*) and a maleylacetate reductase gene (*macA*). In total, strain 1CP encodes seven MacA homologs, whereby two copies of each *macA* gene are located in repeat regions (Table 1). One of the maleylacetate reductases (*clcE2*) is located close to *pheA1*(1) {I104} and in proximity to the second chlorocatechol degradation gene cluster {I103}. The fourth catechol 1,2-dioxygenase (*catA4* {I105}) is encoded in this gene cluster, which has not been described before. Finally, a nitrophenol monooxygenase is encoded on this plasmid {I107}.

**Fig 2:**
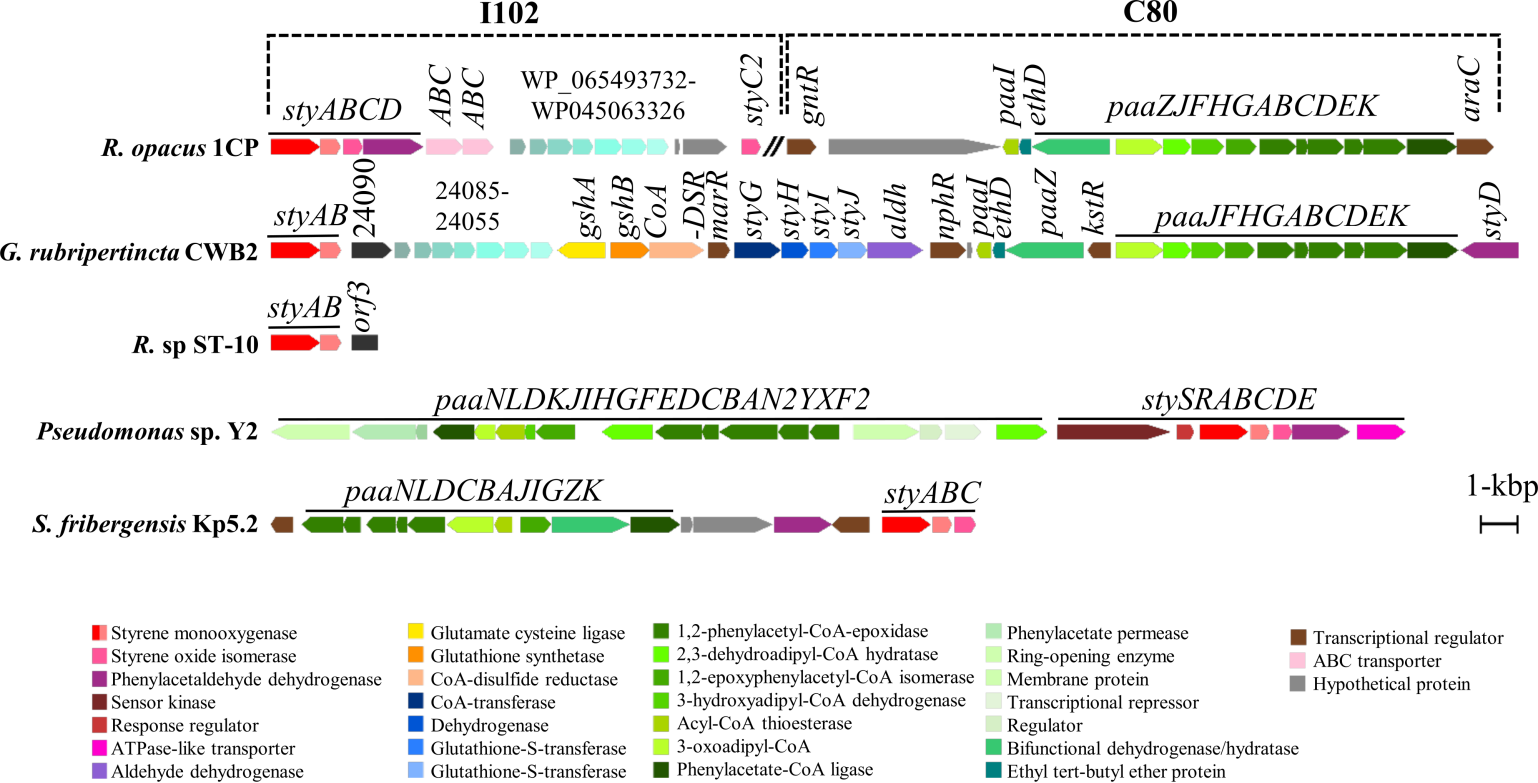
Styrene degradation gene cluster of *Rhodococcus opacus* 1CP and other styrene-degrading microorganisms. Annotations are visualized by a color code, and the location of clusters in the 1CP genome (chromosome or plasmid) is indicated.

Strain 1CP was reported to grow on styrene as a carbon and energy source (74). The degradation of styrene is initiated by a group E flavin monooxygenase (39, 88). Notably, strain 1CP contains three homologs of these enzymes, two of which are encoded next to each other in a single gene cluster {C46}. The third one is located in a well-known gene operon for styrene degradation, *styABCD* {I102} (Fig. 2). The typical arrangement with monooxygenase (*styA/B*), isomerase (*styC*), and dehydrogenase (*styD*) is conserved in many strains. However, the cluster of strain 1CP contains a second isomerase *styC2*, which has not been observed in other strains. Additionally, an insertion of seven hypothetical genes was found similar to the one in the styrene degradation cluster of *Gordonia rubripertincta* CWB2 (Fig. 2). Furthermore, the gene cluster for the lower degradation pathway of the central intermediate phenylacetic acid {C80} resembles that of strain CWB2. These striking similarities suggest possible horizontal gene transfer (89).

The function of the small plasmid pR1CP2 is difficult to determine. It contains only about 65 protein-coding genes, most of which are annotated as hypothetical proteins. However, COG analysis suggests that many of them are associated with replication, recombination, and repair of DNA (COG category L in Table S4). The list of secondary metabolite gene clusters is given in Table S6. Notably, a siderophore biosynthesis gene cluster identical to rhodochelin biosynthesis in *R. jostii* RHA1 is present (90). Earlier, 1CP was reported to produce trehalose dinocardiomycolates when grown on *n-*alkanes (41). The biosynthesis genes for the trehalose precursor (*otsAB* or *treYZ*) are distributed across the chromosomes: *ostA* {C21, C49}, *otsB* (WP_065488964 and WP_065491071), and *treYZ* (WP_065491651 and WP_005563003).

### Construction and growth of pheA1 knock-out mutants

Strain 1CP contains three phenol hydroxylases, *pheA1* (1–3). Double-crossover recombination using the pK18mobSacB vector and the *sacB* counter-selection system (91) successfully produced the knock-out mutants. After conjugation and selection for kanamycin resistance and tolerance to sucrose, colonies were verified by PCR (47). Double- and triple knock-out mutants were obtained by successive homologous conjugation of the wild-type strain using two or three plasmid constructs, respectively. Thus, all possible combinations of *pheA1* knock-outs were achieved in seven mutant strains. The mutant containing all three knock-outs was designated as 1CP^Δ123^, and the wild-type as 1CP^WT^.

Determination of the growth rates of the deletion mutants on phenols was strongly hampered by the high toxicity of certain derivatives, especially chlorophenols. Cultivation on these substrates was usually performed by repeated addition of small substrate aliquots (<0.5 mM) over long time periods, as rapid cell death may occur otherwise. In combination with the tendency of rhodococci to undergo morphological changes during periods of starvation, classical turbidity measurements are unreliable. However, phenol and *p-*cresol can be fed up to 2 mM, yielding a sufficient increase in OD_600_ and can serve as substrates to characterize the knock-out mutants.

1CP^WT^ showed doubling times of 225 min and 405 min when grown on phenol and *p*-cresol, respectively, consistent with the previously reported doubling time of 390 min for growth on *p*-cresol (82). None of the single knock-out mutants showed significant changes in growth rates, whereas noticeable differences were observed in double knock-out mutants (Table 2). The reduced growth rates of double knock-out mutants 1CP^Δ12^ (15%) and 1CP^Δ23^ (52%) relative to 1CP^WT^ (100%) when grown on phenol imply that *pheA1*(2) plays a major role in initial phenol degradation. In contrast, the 1CP^Δ123^ could not grow on phenol (Table 2), indicating that all the relevant genes for phenol degradation were disrupted. This observation correlates with transcriptome analysis, in which *pheA1* (1–3) were upregulated upon phenol exposure (Fig. 1).

**TABLE 2:** Growth parameter comparison of the wild-type strain 1CP and the deletion mutants

Growth substrate	Growth rate (%) of 1CP WT	Growth rate (%) of deletion mutants of *Rhodococcus opacus* 1CP^*a*^						
		1CP^Δ1^	1CP^Δ2^	1CP^Δ3^	1CP^Δ12^	1CP^Δ23^	1CP^Δ13^	1CP^Δ123^
µ on phenol (%)	100	94	75	88	15	52	100	0
µ on *p*-cresol (%)	100	107	105	100	80	98	106	53

^*a*^ Compared to that of the wild-type (WT), set to 100%.

On the other hand, 1CP^Δ123^ showed 53% of the growth rate on *p*-cresol relative to the 1CP^WT^ (Table 2), thus suggesting the involvement of an additional oxygenase. This enzyme has hitherto not been found to be induced by phenol or catechol. Its activity might also influence the kinetic growth parameters of other deletion mutants. Since all single and double knock-out mutants showed almost 100% growth rate relative to 1CP^WT^, the slower growth rate of 1CP^Δ123^ on *p*-cresol may reflect limitations by factors other than phenol hydroxylases.

Only in the case of 1CP^Δ12^, a measured relative growth rate of 80% indicates a rate-limiting initial degradation step and thus allows for the subtraction of the fourth oxygenase activity. Interestingly, the resulting relative growth rate of 98% for 1CP^Δ23^ toward *p*-cresol is consistent with the value obtained for the same mutant during growth on phenol (52%), indicating a comparably low influence of *pheA1*(3) on the initial conversion of both substrates. It should be mentioned that the growth of the double knock-out mutant 1CP^Δ23^ and the triple knock-out mutant on *p*-cresol caused a reddish coloration of the medium.

In addition, in contrast to 1CP^WT^, 1CP^Δ123^ was unable to grow on chlorophenol, indicating that the respective hydroxylases were deleted and, hence, degradation via hydroquinone was not possible. Growth on *p-*nitrophenol was neither observed for the wild-type nor the triple knock-out.

### Transcriptome analysis of the wild-type and the triple knock-out mutant

The transcriptome analysis showcases a variety of physiological activities; only the regulatory responses related to aromatic compound degradation are focused here, that is, the response of strain 1CP^WT^ and mutant 1CP^Δ123^ toward the carbon sources, phenol, *p*-cresol, and styrene (Table S7 and S12), with tetradecane as a reference.

Notably, aromatic O-demethylase was induced by both phenol and *p*-cresol (Table 3), but not by styrene. In 1CP^WT^, phenol upregulated three out of seven phenol hydroxylases, which had earlier been described by Gröning et al. (47) (Table 3, Fig. 1), showing that no other phenol hydroxylases were utilized by 1CP during growth on this substrate. However, 3-hydroxycinnamic-acid hydroxylase {C13}, which produces dihydroxylated intermediates, was expressed with phenol (Table 3). Furthermore, the *ortho-*cleavage pathway was upregulated. Four catechol 1,2-dioxygenase genes were found in the genome, of which two had been described earlier (*catA* {C65} and *catA2* {C75}). Interestingly, the third *catA3* {C67} was also upregulated on phenol, while the fourth *catA4* {I105} was not. The protocatechuate degradation cluster was upregulated with phenol (Table 3), implying the possible metabolization of 3-oxoadipate enol-lactone, formed by *catC*, to succinyl-CoA and acetyl-CoA. None of the catechol 2,3-dioxygenases were upregulated with phenol, except one extradiol dioxygenase {C54} in strain 1CP^WT^. Chlorocatechol degradation clusters ({I97} and {I103}) were either not upregulated or only slightly upregulated with phenol or *p*-cresol in 1CP^WT^ or 1CP^Δ123^, respectively, indicating that they were not involved in phenol or *p*-cresol degradation.

**TABLE 3:** Genes/gene clusters involved in aromatic compound degradation that are differently regulated in 1CP and mutants upon different substrates^*a*^

Gene description	Cluster	WT^TvsS^	WT^TvsP^	∆123^TvsC^	WT vs ∆123^C^
Genes/gene clusters encoded on the chromosome					
Hydrolase cluster	•	-	-	-	(+)
Catechol 2,3-dioxygenase (P17296 - id. 30%)	•	+	-	-	-
Catechol 2,3-dioxygenase (P31003 - id. 47%)	•	+	-	+	+
4-Nitrophenol 2-monooxygenase (NphA1/A2)	•	+	-	+	+
n.a	•	-	+	-	-
N-ethylmaleimide reductase	•	-	+	-	-
Beta-oxidation gene cluster	•	-	-	-	(+)
Superoxide dismutase	•	-	+	+	-
LLM class F420-dependent oxidoreductase	•	-	+	+	-
Glyoxalase/bleomycin resistance/dioxygenase family protein	•	-	+	-	-
Fatty oxidation complex	•	-	-	-	(+)
LLM class flavin-dependent oxidoreductase	ᵒ	-	+	+	-
3-Hydroxycinnamic acid hydroxylase (MhpA_A0KE38 - id. 34%)	•	-	+	-	-
BVMO containing gene cluster	•	-	-	-	(+)
Catalase	•	-	+	-	-
Iron-dependent extradiol dioxygenase (HsaABCD)	•	+	-	-	-
Ectoine hydroxylase	•	-	+	-	-
Cytochrome P450	ᵒ	+	-	-	-
Cytochrome P450 and LLM F420-oxidoreductase	•	+	-	-	-
Nitronate monooxygenase	•	+	-	-	(+)
Trehalose-6-phosphate synthase (OtsA2)	ᵒ	-	-	-	-
Organic hydroperoxide resistance protein	•	-	+	-	-
Nitrilotriacetate monooxygenase	•	-	+	-	-
Pentachlorophenol 4-monooxygenase (PcpB_P42535 - id. 34%)	•	-	(+)	-	-
Glyoxalase/bleomycin resistance/dioxygenase family protein	ᵒ	-	+	+	-
NAD(P)/FAD-dependent oxidoreductase (uncharacterized)	•	-	+	-	-
YceI family protein	•	(+)	+	+	-
Cytochrome P450	ᵒ	+	-	-	(+)
Catalase/peroxidase HPI	•	-	+	+	-
n.a	•	-	+	+	+
ABC transporter	•	+	-	-	-
UDP-glucose metabolism	•	-	+	+	-
Benzoate transporter	ᵒ	-	+	-	-
UDP-glucose pyrophosphorylases (RoGalU2)	•	-	+	-	-
Phenylalanine–tRNA ligase	•	-	+	-	-
Ergothioneine biosynthesis	•	-	+	-	-
Catalase	ᵒ	-	-	+	+
NADH:flavin oxidoreductase lipid metabolism	•	-	+	(+)	(+)
BVMO containing gene cluster	•	-	-	-	-
Methanol:N,N-dimethyl-4-nitrosoaniline oxidoreductase	•	-	-	+	+
Mycofactocin biosynthesis	•	-	-	+	+
Stress response membrane protein	ᵒ	+	-	-	(+)
Propanoyl-CoA carboxylase	•	-	+	+	-
Toxin-antitoxin system HicB family antitoxin	ᵒ	+	-	-	+
Beta-oxidation gene cluster	•	-	-	-	-
Indole monooxygenase (IndA1/A2B)	•	-	-	-	(+)
Riboflavin synthase	•	-	+	-	-
Trehalose dinocardiomycolates	•	-	+	+	-
Trehalose-6-phosphate synthase (OtsA1)	ᵒ	-	-	-	-
Catecholic siderophore biosynthesis NRPS	•	-	+	+	-
3-Hydroxypropionyl-CoA dehydratase	•	-	-	+	+
Additional meta pathway enzymes	•	-	+	+	-
Baeyer-Villiger monooxygenase (A7HU16 - id. 41%)	•	(+)	-	-	-
4,5-DOPA dioxygenase extradiol (DODA_Q70FG7 - id. 42%)	•	-	+	+	-
Propanoyl-CoA carboxylase	•	-	+	+	-
Ene-reductase (OYERo2)	•	-	+	(+)	-
Protocatechuate degradation cluster (PcaJIHGBLRF)	•	(-)	+	-	-
PaaI family thioesterase	•	-	+	-	-
3-Hydroxybenzoate 6-hydroxylase (Q8NLB6 - id. 53%)	ᵒ	-	-	-	-
FAD-dependent oxidoreductase	ᵒ	+	-	-	+
LLM class flavin-dependent oxidoreductase	•	-	+	-	-
n.a	•	-	+	-	-
LLM class flavin-dependent oxidoreductase	•	-	+	+	-
Cytochrome P450	ᵒ	+	+	+	-
Catechol degradation cluster (CatABC)	•	+	+	+	-
Glucose-6-phosphate dehydrogenase	•	-	+	+	+
Catechol 1,2-dioxygenase (CatA3_homolog id 33%)	•	-	+	+	-
Phenol hydroxylase [PheA1(2)]	•	+	+	+	+
Aromatic O-demethylase (P0DPQ7 - id. 79%)	•	-	+	+	+
Benzoate 1,2-dioxygenase	•	(+)	-	-	(+)
n.a	•	-	+	-	-
PPOX class F420-dependent oxidoreductase	•	-	+	-	-
Extradiol dioxygenase and Phenylacetone monooxygenase	•	-	-	+	+
LLM class F420-dependent oxidoreductase	•	-	+	-	-
Phenol hydroxylase [PheA1(3)]/Catechol 1,2-dioxygenase (CatA2)	•	-	+	+	+
Putative 2-hydroxy-6-oxononadienedioate hydrolase	•	+	-	+	+
Alkane 1-monooxygenase/4-Hydroxybenzoate 3-hydroxylase	•	-	-	-	-
LLM class flavin-dependent oxidoreductase	•	-	+	-	-
Anthranilate 1,2-dioxygenase	•	+	-	-	-
Phenylacetic acid degradation cluster	•	+	-	-	-
4-Carboxymuconolactone decarboxylase (PcaC_P20370 - id. 36%)	ᵒ	-	-	-	-
beta oxidation/caffeoyl CoA Lipid metabolism	•	+	-	-	-
Cyclopentanone 1,2-monooxygenase (CpnB_Q8GAW0 - id. 47%)	•	+	-	-	-
Aliphatic amidase (AmiE)	•	+	-	-	-
Cyclopentanone 1,2-monooxygenase (CpnB_Q8GAW0 - id. 47%)	•	+ -	-	-	(+)
Phenylacetone monooxygenase (PamO_Q47PU3 - id. 45%)	•	+	-	-	+
Alkyl hydroperoxide reductase	•	-	+	-	-
Arsenate resistance	•	-	+	-	-
Phenylacetone monooxygenase (PamO_Q47PU3 - id. 52%)	•	+	-	-	-
Terephthalate 1,2-dioxygenase	•	-	+	-	-
Aromatic ring-hydroxylating dioxygenase	•	-	-	-	(+)
Maleylacetate reductase (MacA)/Aromatic ring dioxygenase	•	-	-	-	(+)
ParAB plasmid partitioning and chromosome segregation	•	+	+	-	-
Genes/gene clusters encoded on the plasmid pR1CP1					
Catalase/peroxidase HPI	•	-	+	(+)	-
Aromatic ring-hydroxylating dioxygenase subunit alpha	•	-	-	+	+
Alpha/beta hydrolase	•	-	(+)	+	(+)
4-Chlorocatechol degradation cluster (ClcDARB) {R22} and MacA	•	-	(+)	(+)	-
Glyoxalase/bleomycin resistance/dioxygenase family protein	•	-	+	-	-
Vanillate/3-O-methylgallate O-demethylase	•	+	-	-	-
2,4,5-Trichlorophenol monooxygenase (TftD_O87009 - id. 60%)	•	-	-	-	-
Maleylacetate reductase (MacA_O84992 - id. 100%)	ᵒ	-	-	-	-
Styrene degradation gene cluster	•	+	-	-	-
Chlorocatechol degradation cluster (ClcA2D2B2F)	•	-	-	-	(+)
Phenol hydroxylase [PheA1(1)] and MacA2 (ClcE2)	•	+	+	+	+
Catechol 1,2-dioxygenase (CatA4)	•	-	-	+	+
Azoreductase (AzoRo)	ᵒ	-	-	-	(+)
4-Nitrophenol 4-monooxygenase/4-Nitrocatechol 2-monooxygenase and MacA	•	-	-	-	-

^*a*^ T, tetradecane; C, p-cresol; S, styrene; P, phenol. Genes analyzed in the transcriptional study are listed under “Gene description.” Upregulation and downregulation are denoted by + and -, respectively, while upregulation and downregulation of partial gene clusters or those with insignificant a-value and m-value are given as (+) and (-), respectively. • represents gene cluster, and ○ represents single gene. n.a represents no annotation, while grey highlighting represents an adjacent gene cluster. The respective accession numbers, gene lengths, and locations are provided in Data Set S1.

A clear shift in transcription pattern was observed for 1CP^Δ123^ when grown on *p*-cresol (Table S13). Even though *pheA1* (1–3) were denoted as upregulated (Table 3), they were not functional since they had been knocked out. However, a fourth phenol hydroxylase {C4} (homolog to 4-nitrophenol 2-monooxygenase) was upregulated to take over the initial attack (Table 3). Additionally, one catechol 2,3-dioxygenase {C3} and an extradiol dioxygenase {C73} were upregulated that could initiate *meta*-cleavage on the aromatic ring. Notably, several *meta*-route representatives were found to be upregulated in 1CP^Δ123^ (Table 3). However, many of the *ortho*-pathway enzymes were also upregulated, including *catA3,* which was not in 1CP^WT^. In contrast, the protocatechuate degradation cluster was downregulated in 1CP^Δ123^ with *p*-cresol (Table 3).

**Fig 3:**
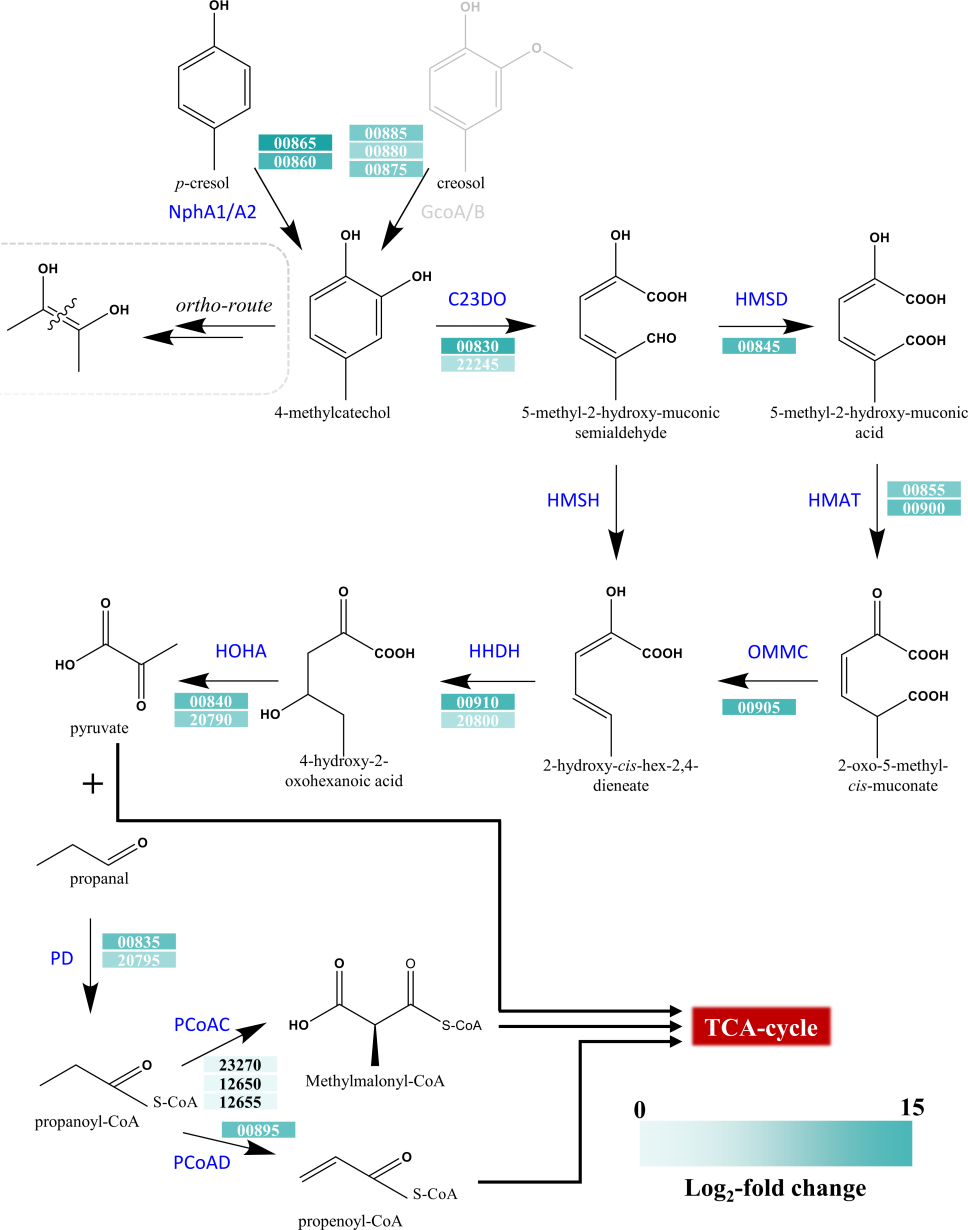
Postulated degradation pathway of *p*-cresol in mutant 1CP^Δ123^ supported by transcriptome data (cyan shading). The initial hydroxylation is catalyzed by a predicted *p*-nitrophenol monooxygenase, showing homology to NphA1/A2 from *Rhodococcus* sp. PN1 (see Fig. 1 and Fig. S6) (92). Interestingly, genes (00875, 00880, 00885) showing homology to the cytochrome P450 aromatic O-demethylase GcoA/B are also upregulated during *p*-cresol exposure and are located in the same gene cluster as the other *meta*-cleavage proteins. The respective enzymes are involved in the degradation of various lignin-relevant monomers like guaiacol and creosol (66). C23DO, 4-methylcatechol 2,3-dioxygenase; PD, propanal dehydrogenase; HOHA, 4-hydroxy-2-oxohexanoate aldolase; HMSD, 2-methyl-2-hydroxymuconic semialdehyde dehydrogenase; 4- NphA1/2, nitrophenol 2-monooxygenase/reductase; HMAT, 5-methyl-2-hydroxymuconate tautomerase; PCoAC, propanoyl-CoA-carboxylase; PCoAD, propanoyl-CoA-dehydrogenase; OMMC, 2-oxo-5-methyl-cis-muconate decarboxylase; HHDH, 2-hydroxyhexa-2,4-dienoate hydratase; HMSH, 2-hydroxymuconate semialdehyde hydrolase.

The 1CP^WT^ and 1CP^Δ123^ cells grown on a small scale (100 mL) with 2 mM *p*-cresol showed similar growth rates (Fig. S5). The specific activities of dioxygenases (Table S8, Fig. S5) showed that 1CP^WT^ attacks the catechol via the *ortho*-cleavage pathway mediated by catechol 1,2-dioxygenase, producing *cis,cis* muconic acid. Whereas 1CP^Δ123^ produced 2-hydroxymuconic semialdehyde, indicating the activity of catechol 2,3-dioxygenase following the *meta*-cleavage pathway. Similar results were observed for 4-methyl catechol, a meta-cleavage-derived product of *p*-cresol (Fig. 3), strengthening the argument that 1CP^Δ123^ employs the *meta*-pathway to act on substituted phenols.

In 1CP^WT^, styrene induced the styrene degradation gene cluster *styABCD*, encoded on the plasmid pR1CP1, including several hypothetical proteins, a second copy of styrene oxide isomerase *styC2*, and some downstream genes. Several Baeyer-Villiger, P450, and 4-nitrophenol 2-monooxygenases were also upregulated by styrene. On the other hand, indole monooxygenase (*IndA1/A2B*), known to epoxidize styrene, was not induced by styrene as a carbon source.

### Purification and kinetics of *Ro*SOI1 and *Ro*SOI2

Styrene oxide isomerases (StyC/SOI) are transmembrane proteins that catalyze the conversion of styrene oxide to phenylacetaldehyde. 1CP has previously been described for styrene oxide isomerase activity (*Ro*SOI1), but the role of the second copy of SOI (*Ro*SOI2) was unclear. Therefore, both enzymes were expressed and studied for their activity. The heterologous expression of SOIs in *E. coli* BL21 (DE3) yielded only about 0.34 mg and 0.13 mg of enzymes per liter of expression culture for *Ro*SOI1 and *Ro*SOI2, respectively. It is not uncommon for membrane proteins to have such low yields. The Michaelis-Menten kinetics (Table S9) for both enzymes were investigated by directly monitoring the product phenylacetaldehyde using RP-HPLC. By comparison, *Ro*SOI1, encoded within the styrene degradation gene cluster, showed twice the catalytic efficiency (2.1 × 10^6^ s^−1^ M^−1^) in converting the natural substrate (*R/S*)-styrene oxide, compared to *Ro*SOI2 (1.09×10^6^ s^−1^ M^−1^). Interestingly, *Ro*SOI1 showed a specific activity of about 299 ± 16 U mg^−1^, while *Ro*SOI2 showed 374 ± 13 U mg^−1^, which is closer to the value of 375 U mg^−1^ reported earlier (85) for an enriched SOI membrane fraction in 1CP. Since the difference was minor, the precise role of the two SOIs could not yet be clarified, but from the transcriptome and kinetic data, it is clear that both SOIs are involved in the metabolism of styrene degradation intermediates.

## DISCUSSION

The metabolic versatility of *Rhodococcus* strains has previously been attributed to two main reasons: first, the large genome with one or more plasmids, and second, the presence of redundant genes (83, 84). Both reasons are true for strain 1CP. With a size of about 8.6 Mbp, the 1CP genome is among the largest genomes of *Rhodococcus* compared to the average size of completed genomes of 6.2 Mbp. Recently, the number of omics data and functional studies on *Rhodococcus* strains has rapidly increased, leading to about 900 strains being sequenced and listed on NCBI. Notably, *R. jostii* RHA1 was the first genome sequencing of the genus in 2001. Since then, Zampolli reported 236 genomes belonging to *Rhodococcus* species in 2019 (14). Interestingly, the gene clusters related to degradation of halogenated aromatic compounds and one phenol hydroxylase *pheA1*(1) (47, 85) were encoded on the megaplasmid pR1CP1. Additionally, the styrene degradation gene cluster was also located on the same plasmid and shows homology to the one found in *Gordonia rubripertincta* CWB2 (Fig. 2) (89). Owing to differences in G+C content, the horizontal gene transfer analysis, and the presence of many other aromatic-degrading genes and gene clusters, it is likely that the megaplasmid is of foreign origin. The gene redundancy is illustrated by the presence of seven phenol hydroxylases (Fig. 1), several cytochrome P450-monooxygenases, Baeyer-Villiger monooxygenases, and other hydroxylases; four catechol 1,2-dioxygenases and two catechol 2,3-dioxygenases (among other intradiol and extradiol dioxygenases); and seven maleylacetate reductases (Data Set S1). In addition, some of the degradation gene clusters are located in repeat regions of the plasmid pR1CP1.

Even though strain 1CP was extensively studied for phenolic compound degradation, only the *ortho*-degradation was described for it and also for other rhodococci (93, 94). Recent genomic analysis of 37 rhodococci showed that some do not possess the genes for *meta*-cleavage, while some exclusively prefer the *ortho*-route despite having genes for *meta*-cleavage pathways for phenol (94). This was true for the closest neighbor of the study strain, *Rhodococcus opacus* PD630, which was grown on 1-^13^C-labeled phenol and showed that the strain exclusively chose the *ortho-*cleavage route. The regulation of *orthro*-cleavage genes in 1CP and PD630 is listed in Table S14. Furthermore, the knock-out of a key enzyme in the *ortho*-pathway resulted in the inability to utilize phenol (93).

Similar behavior was observed for strain 1CP from growth and transcriptome analyses. Only three out of seven phenol hydroxylases, *pheA1* (1–3), were upregulated on phenol as published earlier (63), which were also recently found to be induced in germinating 1CP cells after dormancy (95). Phylogenetic analysis supports that only those three were closely related and clustered with well-studied phenol hydroxylases, while the other four grouped themselves among halo- or nitrophenol hydroxylases (Fig. 1). This suggests that such halo- or nitrophenol derivatives activate the hydroxylases other than *pheA1*. Subsequently, the upregulation of catechol degradation cluster *catABC*, catechol 1,2-dioxygenases, the protocatechuate cluster, and some other aromatic compound monooxygenases shows that strain 1CP takes the *ortho*-route. Additionally, no extradiol dioxygenases or catechol 2,3-dioxygenases were upregulated, which clearly demonstrates that strain 1CP exclusively takes the *ortho*-route to degrade phenolic compounds. Loss of one or two phenol hydroxylases was well compensated by 1CP, while the triple knock-out mutant lost the ability to grow on phenol, indicating that phenol does not induce any other phenol hydroxylases. On the other hand, the growth rate of 1CP^Δ123^ was much lower than that of 1CP^WT^ or other mutants in the presence of *p-*cresol, which suggests that *p*-cresol was used more efficiently by the *ortho*-route compared to the *meta*-pathway, or that the latter might already be induced in single and double mutants to compensate for the loss. However, this needs to be experimentally proven. It was described earlier that 1CP^WT^ showed no *meta*-cleavage enzyme when grown on *p*-cresol (82).

The expression of another phenol hydroxylase (Fig. 1) by *p*-cresol and various *ortho*-enzymes in the triple mutant (Table 2) suggests that 4-methylcatechol formed by NphA1 was also funneled into the “classical” intradiol degradation route (82). However, the protocatechuate gene cluster, including *pcaL, pcaIJ,* and *pcaF*, is not upregulated. This leads to the assumption that the respective gene cluster is not induced by *p*-cresol or 4-methylcatechol. It is also possible that none of the *ortho*-pathway metabolites are produced, which could induce the protocatechuate gene cluster. Another possible route for the degradation of *p*-cresol would be the metabolization via a hydroxyl 1,4-benzoquinone, hydroxyquinol, and subsequently maleylacetate (92, 96). This route has been described for nitrophenol degradation in several other rhodococci and *Arthrobacter* species (92, 96–99). The nitrophenol hydroxylases that execute the initial reaction were closely related to the phenol hydroxylases (Fig. 1) and usually encoded together with a regulator and, in some cases, together with a hydroxyquinol 1,2-dioxygenase (Fig. S6). In strain 1CP, both cluster variants can be found {C4, I107}, whereas the latter one is not upregulated under any of the applied conditions (Tables S7 and S12), which suggests that degradation via the quinone does not occur in strain 1CP.

Therefore, it is likely that the mutant 1CP^Δ123^ executes the degradation of *p*-cresol via a *meta*-route initiated by catechol 2,3-dioxygenase {C3}. This hypothesis is supported by dioxygenase activity and the transcriptomic analysis, which show that relevant genes required for the subsequent formation of central metabolic intermediates, such as pyruvate and propenoyl-CoA, were expressed. Interestingly, aromatic O-demethylases were also expressed when *p-*cresol was supplied as substrate. These enzymes are related to lignin breakdown, which might be a hint that the proposed degradation pathway plays a role in the consumption of lignin cleavage products and is induced by *p*-cresol. The interpretation is reinforced by the fact that creosol is derived from lignin and shows high similarity to cresol (100).

The transcriptome study of *R. rhodochrous* EP4 involved in the catabolism of one of the depolymerization products of lignin, alkylphenols, revealed that 4-ethylphenol was achieved via *meta*-cleavage (101). This gene cluster shows high similarity to the one found in 1CP, and the proposed degradation pathway resembles the one from this study (Fig. 3), suggesting that this pathway might play a role in lignin degradation. In contrast to this study, the aromatic O-demethylase (GcoA/B) was not induced in strain EP4. The deletion mutant of catechol 2,3-dioxygenase in strain *R. jostii* RHA1 lost the ability to grow on alkylphenol, whereas the deletion of alkylphenol hydroxylase had no impact on growth but resulted in higher expression of three phenol hydroxylases, similar to the redundant *pheA1* in 1CP. In EP4, the *catABC* cluster is induced, but no *ortho*-cleavage metabolites were detected. In 1CP, during the growth of the strain on *p*-cresol, activities of the *ortho*-enzymes were detected, while those of the catechol 2,3-dioxygenases were not found, indicating that *p*-cresol can be funneled through the *ortho*-route. This could mean that alkylphenols with larger substituents cannot be metabolized via the *ortho-*route, but only those with short side chains.

A third possibility is to attack the side chain of aromatic hydrocarbons, as in the well-known breakdown of styrene in strain 1CP (74). In this context, we were able to support previous suggestions regarding the styrene degradation cluster {I102} located on the plasmid of strain 1CP. Further, it seems to be the case that this cluster is larger than expected. Besides the genes known to be required for degradation (*styABCD*), two transporters, several hypothetical membrane proteins, and a second styrene oxide isomerase, *styC2*, are upregulated (Fig. 2; Data Set S1). Also, the genes for the degradation of the central intermediate phenylacetic acid were upregulated {C80}, which completes the picture of styrene degradation in strain 1CP. Interestingly, also several P450 monooxygenases are induced during growth on styrene (Table 3). These enzymes are known to be able to epoxidize styrene in humans and might therefore also promote styrene activation in strain 1CP.

So far, only one styrene-specific ABC-transporter (StyE) has been described from *Pseudomonas* (102), but aligning this one to the ABC-transporter found in strain 1CP failed to identify similarity. Thus, the role of these two ABC transporters in styrene import can only be postulated. The same is true with the set of seven hypothetical proteins that show very high similarity to those recently found in a styrene degradation cluster of *Gordonia rubripertincta* CWB2 (89). In that strain, the degradation of styrene occurs over glutathione. The finding that this set of hypothetical proteins was induced in two different strains and styrene degradation clusters, respectively, strongly suggests a role in styrene metabolization. However, so far, the only known characteristics are that five of these proteins are likely to have a transmembrane section. Thus, further research is needed to clarify this.

Another interesting observation is that the second styrene oxide isomerase (*styC2*) was also induced during styrene exposure. Based on the kinetic data, StyC1 (*Ro*SOI1) is twice as effective as StyC2 (*Ro*SOI2) with respect to catalytic efficiency and substrate affinity for styrene oxide. This suggests that *Ro*SOI1 is the primary SOI, while the role and nature of *Ro*SOI2 are still unclear. Further studies on substrate scope, other biochemical characterization, or transcriptome analysis might be needed for the second copy to determine its exact role.

In conclusion, we report the complete genome of strain 1CP and compare the genome and transcriptome data when grown on aromatic compounds, along with various previous studies. This helped to trace the three main pathways of aromatic compound degradation— *ortho*-, *meta*-, and side chain—completing the picture that was begun in numerous earlier studies on this versatile strain, *Rhodococcus opacus* 1CP.

## MATERIALS AND METHODS

### Cultivation of strains

For scanning electron microscopy, the cells of 1CP were grown and treated, fixed, and scanned as described in Oelschlägel et al. (75). Similarly treated cells were transferred onto a microscopic slide containing 1.5% (wt/vol) agarose coating, covered with a coverslip, and subjected to bright field microscopy. Fed-batch cultivation of *Rhodococcus opacus* 1CP wild-type was performed at 30°C in mineral medium (103) at pH 7.2, containing phenol (24–47 mM), 4-chlorophenol (4CP) (23 mM), *p*-cresol (21–25 mM), or benzoate (25 mM). Final concentrations were achieved by portion-wise addition of the carbon source over 4–5 days. Depending on the desired amount of biomass, growth was performed in 1 L and 3 L baffled shaking flasks (130 rpm constant shaking) or a 5 L glass fermenter (ADI 1030 biocontroller, Applikon, Schiedam, the Netherlands). Cells were harvested by centrifugation (15,000 ×*g*), washed with 50 mM K/Na-phosphate buffer (pH 7.2), shock-frozen in liquid nitrogen, and stored at –80°C until used.

For growth experiments of the hydroxylase-deficient mutants and the wild-type, 10 mL precultures were cultivated in 100 mL baffled Erlenmeyer flasks containing the above mineral medium and 4 mM benzoate as the C-source (130 rpm, 30°C). Twenty-four hours later, the cultures were centrifuged (15,000 × *g*), washed with 25 mM K/Na-phosphate buffer, and resuspended in 40 mL of the same phosphate buffer. Then, 100 mL mineral medium, containing either 2 mM phenol or 2 mM *p*-cresol, was inoculated with these washed cells to a final OD_600_ of 0.1. Cultivation took place in 1 L shaking flasks at 30°C (130 rpm). Growth rates μ (µ = (lnx_1_ − lnx_0_) / (t_1_ – t_0_)) and doubling times were calculated from the exponential phase. Each experiment was carried out in triplicate. *E. coli* DH5α, *E. coli* BL21-CodonPlus-RP, and *E. coli* S17-1 were grown in Luria-Bertani (LB) medium (104) at 20°C, 30°C, or 37°C. When appropriate for selection, the medium was supplied with ampicillin (100 µg mL^−1^) and/or chloramphenicol (50 µg mL^−1^). LB plates used for conjugation contained nalidixic acid (30 µg mL^−1^) and kanamycin (20 µg mL^−1^). To select for allelic exchange by homologous recombination, 0.5× LB plates were supplemented with 10% sucrose. OD_600_ was measured in quartz-glass cuvettes (Cary50 spectrophotometer, Varian).

### Genome sequencing, assembly, and annotation

The genomic DNA of *Rhodococcus opacus* 1CP was isolated as described before for other actinobacteria (89). DNA sequencing of small genomic fragments was performed as described elsewhere (105). The library preparation, genome sequencing, assembly, annotation, and subsequent analysis were performed as described previously (89). Sequences were analyzed with the Lasergene 99 program package (v4.05, DNASTAR) and the Staden package (version 1.6.0) (106), or by using the RAST platform (107), and compared with the non-redundant database provided by the National Center of Biotechnology Information (NCBI), using BLAST (108). Alignment of peptide sequences and phylogenetic analysis was performed with MEGAX (109) using the default parameters.

### DNA preparation, *in vitro* manipulation, sequencing, and analysis

Plasmid DNA from clones was isolated using the FlexiPrep Kit (GE Healthcare Europe GmbH). Genomic DNA from *Rhodococcus opacus* 1CP was prepared as described previously (49). DNA digestion with restriction endonucleases (MBI Fermentas, Germany), electrophoresis, and ligation with T4 DNA ligase (MBI Fermentas) were performed according to standard protocols (104) and the supplier’s instructions. DNA was eluted from agarose gels by means of the EasyPure Kit (Biozym) and the GeneClean kit (Qbiogene GmbH, Heidelberg, Germany). Transformation of *E. coli* strains was performed according to Inoue (110).

### Construction of pheA1 knock-out mutants

Three different plasmids were constructed to inactivate the three *pheA1* genes [pheA1(1), pheA1(2), and pheA1(3)] (Table S10). In all cases, pK18mobSacB (111) served as backbone. The plasmids were conjugated via *E. coli* S17-1 into *Rhodococcus opacus* 1CP. By successive conjugations, double and triple knock-out mutants, respectively, were constructed. Integration of plasmid and allelic exchange by homologous recombination was verified by PCR.

The plasmid pROPH3 was digested with *Bam*HI and *Eco*RI. A 1.47 kb DNA fragment was isolated and purified by means of gel electrophoresis. It was subsequently ligated into the multiple cloning site of pK18mobSacB and designated as pK18A1-3E. The fragment covers 893 bp of the pheA1 gene, 542 bp of the putative regulator gene thcR, and the interregion.

To interrupt the *pheA1(2*) gene, a 333 bp large *Dra*II fragment of pROPH3 was inserted in the solely *Dra*II restriction site of pK18A1-3E, which cuts inside the *pheA1* gene. The construct with the disrupted *pheA1* gene was designated as pK18A1-3E91. The construct was conjugated via *E. coli* S17-1 into *R. opacus* 1CP. First crossover was validated by means of PCR. The colony that exhibited the expected PCR amplicon was designated as *R. opacus* 1CP E7.

For the selection of the second crossover, an overnight culture of *R. opacus* 1CP E7 in LB was spread on 0.5× LB plates containing 10% of sucrose. Only clones that had lost the pK18 insertion could grow on these plates. To distinguish between revertants and mutants, the colonies were checked by PCR. Additionally, mutants were checked by DNA sequencing. The mutant with the disrupted *pheA1(2*) gene was designated as *R. opacus* 1CP E740.

For the knock-out of the *pheA1(1*) gene, the plasmid pK18A1-1EN1X4 was constructed. Therefore, the whole 3.7 kb *Eco*RI fragment was excised from pROPH1-2 and ligated into pK18mobSacB, resulting in pK18ROPH1-2c. This plasmid was digested by *Not*I and the 1.9 kb fragment religated (pK18A1-1EN). In order to inactivate *pheA1(1*), pK18A1-1EN was digested by *Xho*I and a 344 bp *Xho*I fragment of pROPH8 was inserted.

To disrupt *pheA1(3*) of pROPH8, the plasmid pK18A1-8P1E2X1 was constructed. In this case, pROPH8 was digested with *Pst*I, and the 2.1 kb fragment was ligated into pK18mobSacB. The resulting plasmid (pK18A1-8P1) was cut with *Eco*RI, and the 1.6 kb fragment was religated (pK18A1-8P1E2). This fragment covers a catechol 1,2-dioxygenase and the last 650 bp of *pheA1(3*). To interrupt the reading frame of *pheA1,* a 344 bp *Xho*I fragment of pROPH8 was ligated into the *Xho*I site of pK18A1-8P1E2.

Transfer of the plasmid into *Rhodococcus opacus* 1CP and verification of the first and second crossover, respectively, was carried out as described above.

### Transcriptome sequencing and analysis

For the cultivation of cells for tetradecane, phenol, and *p*-cresol studies, the procedure was as follows. Precultures of *R. opacus* 1CP^WT^ and *R. opacus* 1CP ^Δ123^ were grown in 20 mL minimal medium (75) containing 4 mM benzoate as sole carbon source at 30°C under continuous shaking. After 4 days, the cultures were fed with 5 mM benzoate. After 5 and 7 days of growth, 2 mM of the above-mentioned carbon sources were added. After 8 days, cultures were divided into four shaking flasks for each substrate containing 100 mL of fresh minimal medium and 2 mM of the respective carbon source. Afterward, the cultures were fed twice daily with 2 mM of the respective carbon source. This was done for 3 days before harvesting, according to the protocol described earlier (89). In the case of tetradecane, the purified RNA samples were pooled before sequencing.

For the cultivation of 1CP^WT^ cells for styrene transcriptome studies, the preparation followed earlier studies for the actinobacterium *Gordonia rubripertincta* CWB2 (89). RNA quality analysis and sequencing were performed as described earlier (89). The reads were mapped on the reference strain *R. opacus* 1CP (CP009111, CP009112, and CP009113) using Bowtie2 (112) with standard settings. Visualization and data analysis were performed using the software ReadXplorer 2.2.3 (113). Differential gene expression analysis was based on normalized read counts using TPM values (transcripts per million) of coding sequences (CDSs) calculated by ReadXplorer 2.2.3. The signal intensity value (a-value) was calculated as 0.5 × (log_2_ TPM condition A + log_2_ TPM condition B) of each CDS, and the signal intensity ratio (m-value) was calculated as the difference of (log_2_) TPM. CDS with m-values of higher/equal than ± 0.58 and a padj lower than 0.05 were considered to be differentially transcribed.

### Dioxygenase activity of 1CP^WT^ and 1CP^∆123^ grown on *p*-cresol

The cells of 1CP^WT^ and 1CP^∆123^ were grown in 100 mL mineral medium with 2 mM *p*-cresol as sole carbon source, as mentioned earlier. The cells were washed with 50 mM potassium phosphate buffer, pH 7.5. The cells were disrupted by ultrasonication (seven cycles of 30-second pulses followed by 60-second pauses, 30% output), and the cell-free lysate was prepared by centrifugation at 9,000 × *g* for 40 min at 4°C, which served as the enzyme solution. The protein concentration of the cell-free lysate was determined using the Bradford assay with BSA as the standard. The activity assays were performed in a 96-well plate with a total volume of 250 µL of reaction mixture, containing 200 µL of the above-mentioned buffer and 50 µL of enzyme solution. The C12DO activity was measured by monitoring the formation of *cis,cis*-muconic acid at 260 nm (ε_260_ = 16,800 M^−1^·cm^−1^) using catechol as the substrate. For C23DO activity, the changes were observed by monitoring the formation of 2-hydroxy muconic semialdehyde at 375 nm (ε_375_ = 33,000 M^−1^·cm^−1^) and 2-hydroxy-5-methyl muconic semialdehyde at 382 nm (ε_382_= 28,100 M^−1^·cm^−1^) using catechol and 4-methyl catechol as substrates, respectively. The specific activities were calculated using their respective molar extinction coefficient and expressed as nmol min^−1^ mg^−1^ (mU mg^−1^). The assay was performed in triplicates with both biotic and abiotic controls (114).

### Heterologous expression, purification, and quantification of *Ro*SOI1 and *Ro*SOI2

The codon-optimized SOIs were cloned into pET28a(+) vector with *N-*terminal His_6_ tag and transformed into *E. coli* BL21 (DE3) expression system. The SOIs were cultivated in 2.8 L Erlenmeyer flasks containing 1 L of autoinduction medium and purified as described in Kumaran et al. (115). The protein concentration was determined using the extinction coefficient of the heme at 420 nm, ε_420_ = 115 mM^−1^·cm^−1^ (116).

### Michaelis-Menten kinetics of SOIs

The Michaelis-Menten kinetics of SOIs for its natural substrate, (*R/S*)-styrene oxide, were studied using sampling method and measured by RP-HPLC. The enzyme assay was performed in a 1.5 mL glass vial at 25°C, 1,000 rpm. The assay, sample preparation for RP-HPLC measurements, and analytics were performed as described in Kumaran et al. (115). The initial velocities with respect to substrate concentration were plotted against each other to determine the kinetic parameters, *K_M_*, *k*_cat_, and *V*_max_. All the measurements were performed in triplicates.

## Data Availability

*Rhodococcus opacus* 1CP is available in public culture collections (DSM 46757 and VKM: Ac-2638). All other strains and plasmids used in this study are listed in Tables S10 and S11. A summary of transcriptome sequencing statistics is deposited in DataSet S2. The genome sequence is available via the GenBank accessions CP009111 to CP009113 and BioProject PRJNA253567. The RNA-seq data as well as the raw reads are available via GEO accession GSE294731. Accession numbers of relevant genes and proteins of this study can be found in the respective sections of the paper as well as the supplemental material. The complete list of gene/gene clusters identified in strain 1CP is provided in Data Set S1, and the transcriptome sequencing statistics and annotations are provided in Data Sets S2 and S3.

## SUPPLEMENTAL MATERIAL

Supplemental material aem.00045-26-s0001.docx Tables S1 to S14; Fig. S1 to S6.

Data Set S1 aem.00045-26-s0002.xlsx Genes/gene clusters differently regulated in strain 1CP upon treatment with different aromatic compounds.

Data Set S2 aem.00045-26-s0003.xlsx Transcriptome annotation of 1CP grown on styrene (treatment) vs. *p*-cresol (control).

Data Set S3 aem.00045-26-s0004.xlsx Transcriptome statistics of 1CP^∆123^ grown on p-cresol (treatment) and tetradecane (control).

ASM does not own the copyrights to Supplemental Material that may be linked to, or accessed through, an article. The authors have granted ASM a non-exclusive, world-wide license to publish the Supplemental Material files. Please contact the corresponding author directly for reuse.
