# Supplementary material for: Whole-genomic and transcriptomic analyses elucidate p-cresol and styrene degradation metabolism in Rhodococcus opacus 1CP
Source: Appl Environ Microbiol. 2026 Mar 27;92(4):e00045-26. doi: 10.1128/aem.00045-26 (PMC13101518; doi:10.1128/aem.00045-26)
Supplement: Supplemental material — Tables S1 to S14; Fig. S1 to S6. [file aem.00045-26-s0001.docx]

**Supplementary materials**

**Whole-genomic and transcriptomic analyses elucidate *p*-cresol and styrene degradation metabolism in *Rhodococcus opacus* 1CP**

Selvapravin Kumaran (0000-0002-0799-9211),^a^ Thomas Heine,^b,^* Janosch A.D. Gröning,^b,§^ Michael Schlömann,^b^ Andreas Albersmeier,^c^ Tobias Busche ([0000-0001-9211-8927](https://orcid.org/0000-0001-9211-8927)),^c,d^ Jörn Kalinowski,^c^ Christian Rückert-Reed ([0000-0002-9722-4435](https://orcid.org/0000-0002-9722-4435)),^c,d^ Lena Schaffert ([0000-0002-2437-1784](https://orcid.org/0000-0002-2437-1784)),^c^ and Dirk Tischler,^a,b^ (0000-0002-6288-2403)^#^

^a^Microbial Biotechnology, Faculty for Biology and Biotechnology, Ruhr University Bochum, Bochum, Germany

^b^Institute of Biosciences, Faculty of Chemistry, Physics and Biosciences, TU Bergakademie Freiberg, Freiberg, Germany

^c^Technology Platform Genomics, Center for Biotechnology (CeBiTec), Bielefeld University, Germany

^d^Medical School OWL, Bielefeld opeUniversity, Germany

Running Head: Transcriptome of aromatic compound degrader strain 1CP

^#^Address correspondence to Dirk Tischler, [dirk.tischler@email.de](mailto:dirk.tischler@email.de)

*Present address: Chair of Molecular Biotechnology, Institute of Microbiology, TU Dresden, Dresden, Germany.

^§^Present address: Institute of Microbiology, University of Stuttgart, Stuttgart, Germany.

Selvapravin Kumaran and Thomas Heine contributed equally to this work. Author order was decided by mutual consultation.

**Table S1**. List of studies conducted on *Rhodococcus opacus* 1CP for various applications

| Studies | | | References |
| --- | --- | --- | --- |
| **Degradation** | (Halo-)aromatic compounds | (Halo)catechols | 65, 68-71, 82, 92 |
|  |  | (Halo)phenols | 40, 63, 65, 67, 68, 73, 77, 91 |
|  |  | Benzoates, (Halo)benzoates, (Hydroxy)benzoates, (Methyl)benzoates | 58, 67 |
|  |  | *para*-toluate | 43 |
|  |  | *para*-cresol | 90 |
|  | Dyes | Methyl red | 42 |
|  | (Long-chain) alkanes | e.g. n-tetradecane and n-hexadecane | 44, 50 |
|  | Biosurfactants | Tween 80 | 20 |
|  | Heterotrophic CO_2_ assimilation _­_ | | 45 |
| **Production** | Nocardiomycolic acids |  | 50 |
|  | Biosurfactants | Trehalose lipids | 50, 51, 19 |
|  | Phenylacetic acids | Styrene | 86 |
| **(Stress) response** | Effect of (halo‑)aromatic compounds | on fatty acid production | 41 |
|  |  | on cell and colony morphology, lipid composition and growth manner | 31 |
|  | Dormancy | effects aromatic compound degradation | 32, 33 |
|  | Oxidative stress | Effect on morphological, physiological and biochemical characteristics | 34 |
| **Biotechnology** | Cell immobilization |  | 34, 35 |

**Table S2**. List of substrates that were tested as carbon source for *Rhodococcus opacus* 1CP in agar plate

| **Aminoacids [mM]** |  | **(Aromatic) Carboxylic acids [mM]** |  | **Aromatic hydrocarbons [mM]** |  |
| --- | --- | --- | --- | --- | --- |
| Alanine [4] | + | Gluconic acid [4] | + | Phenol | + |
| Arginine [4] | + | Succinic acid [10] | + | 3-Methylphenol (*m*-cresol) | + |
| Aspartic acid [4] | + | Benzoic acid [10] | + | 4-Methylphenol (*p*-cresol) | + |
| Cysteine [4] | - | 4-Methylbenzoic acid [2] | + | 4-Chlorophenol | + |
| Glutamine [4] | + | 2-Hydroxybenzoic acid [5] | - | 2,4-Dichlorophenol | + |
| Glutamic acid [4] | + | 3-Hydroxybenzoic acid [5] | + | Acetophenone | + |
| Glycine [4] | + | 4-Hydroxybenzoic acid [5] | + | Styrene | + |
| Histidine [4] | - | 2,5-Dihydroxybenzoic acid [200 mg L^-1^] (89) | + | Styrene oxide | + |
| Lysine [4] | - | 3,4-Dihydroxybenzoic acid [2] | + | Phenylacetaldeyde [0.5] | + |
| Phenylalanine [4] | + | 2-Aminobenzoic acid [4] | + | Vanillin [4] | + |
| Proline [4] | + | 3-Aminobenzoic acid [5] | + | 2-Phenylethanol [1] | + |
| Serine [4] | + | 4-Aminobenzoic acid [5] | + | Naphthalene [6.7 g L^-1^] | - |
| Tryptophan [4] | - | Phthalic acid [4] | + | Phenanthrene [6.7 g L^-1^] | - |
| Tyrosine [4] | + | trans-Ferulic acid [4] | + | Biphenyl [6.7 g L^-1^] | - |
|  |  | Caffeic acid [4] | + |  |  |
| **Monosaccharides [mM]** |  | trans-Cinnamic acid [4] | + | **Complex media** |  |
| Arabinose [4] | - | 6-Hydroxynicotine acid [5] | + | LB | + |
| Fructose [4] | + | Nicotinic acid [5] | + | dYT | + |
| Galactose [4] | + | Phenylacetic acid [10] | + |  |  |
| Glucose [4] | + | Vanillic acid [10] | + |  |  |
| Rhamnose [4] | - | 2-Chlorobenzoic acid [4] | - |  |  |
| Ribose [4] | + | 3-Chlorobenzoic acid [4] | + |  |  |
| Xylose [4] | - | 4-Chlorobenzoic acid [4] | - |  |  |
|  |  | Pyruvic acid [4] | + |  |  |
| **Disaccharides [mM]** |  | cis-cis-Muconate [3] | + |  |  |
| Cellobiose [4] | - |  |  |  |  |
| Lactose [4] | + | **Alditols [mM]** |  |  |  |
| Maltose [4] | + | Sorbitol [4] | + |  |  |
| Saccharose [4] | + | Mannitol [4] | + |  |  |
|  |  |  |  |  |  |
| **Alkane [mL L^-1^]** |  | **Steroids** |  |  |  |
| N-Decane [13] | + | Cholesterol | + |  |  |
| N-Dodecane [13] | + |  |  |  |  |
| n-Tetradecane [13] | + |  |  |  |  |
| n-Hexadecane [13] | + |  |  |  |  |

+ = growth; - = no growth

**Table S3**. Genome statistics of *Rhodococcus opacus* 1CP

| Attribute | Chromosome | Plasmid (pR1CP1) | Plasmid (pR1CP2) |
| --- | --- | --- | --- |
| Accession | CP009111 | CP009112 | CP009113 |
| Sum of contig length (bp) | 7,687,653 | 885,383 | 64,499 |
| G + C content (%) | 67.4 | 64.1 | 63.7 |
| Protein coding genes | 7,380 | 881 | 65 |
| Average gene length (bp) | 968 | 816 | 858 |
| Coding percentage (%) | 93 | 81 | 87 |
| tRNA genes | 59 | 2 | - |
| rRNA genes | 12 | - | - |

**Table S4**. Assignment of genes of *Rhodococcus opacus* 1CP to clusters of orthologous genes (COG) categories by IMG.

| Category and function | | Predicted proteins assigned to the COG category | |
| --- | --- | --- | --- |
|  |  | numbers | ratio (%) |
| A | RNA processing and modification | 1 | 0.01 |
| B | Chromatin structure and dynamics | 1 | 0.01 |
| C | Energy production and conversion | 513 | 6.78 |
| D | Cell cycle control, cell division, chromosome partitioning | 46 | 0.61 |
| E | Amino acid transport and metabolism | 656 | 8.67 |
| F | Nucleotide transport and metabolism | 129 | 1.71 |
| G | Carbohydrate transport and metabolism | 455 | 6.02 |
| H | Coenzyme transport and metabolism | 418 | 5.53 |
| I | Lipid transport and metabolism | 675 | 8.93 |
| J | Translation, ribosomal structure and biogenesis | 231 | 3.05 |
| K | Transcription | 741 | 9.80 |
| L | Replication, recombination and repair | 202 | 2.67 |
| M | Cell wall/membrane/envelope biogenesis | 227 | 3 |
| N | Cell motility | 17 | 0.22 |
| O | Posttranslational modification, protein turnover, chaperones | 200 | 2.64 |
| P | Inorganic ion transport and metabolism | 495 | 6.55 |
| Q | Secondary metabolites biosynthesis, transport and catabolism | 471 | 6.23 |
| R | General function prediction only | 1029 | 13.61 |
| S | Function unknown | 355 | 4.69 |
| T | Signal transduction mechanisms | 273 | 3.61 |
| U | Intracellular trafficking, secretion, and vesicular transport | 42 | 0.56 |
| V | Defense mechanisms | 165 | 2.18 |
| W | Extracellular structures | 8 | 0.11 |
| X | Mobilome: prophages, transposons | 213 | 2.82 |
|  | Not in COG | 2189 | 26.54 |

**Table S5**. Assignment of genes of *Rhodococcus opacus* 1CP to subsystems by RAST

|  | Chromosome | | pR1CP1 | | pR1CP2 | |
| --- | --- | --- | --- | --- | --- | --- |
| Subsystem coverage | 23 % |  | 7 % |  | 3 % |  |
| Description | Value | % | Value | % | Value | % |
| Cofactors, vitamins, prosthetic groups, pigments | 287 | 9.9 | 8 | 10.4 | 0 | 0.0 |
| Cell wall and capsule | 44 | 1.5 | 0 | 0.0 | 0 | 0.0 |
| Virulence, disease and defense | 45 | 1.6 | 1 | 1.3 | 0 | 0.0 |
| Potassium metabolism | 15 | 0.5 | 0 | 0.0 | 0 | 0.0 |
| Miscellaneous | 49 | 1.7 | 2 | 2.6 | 0 | 0.0 |
| Phages, prophages, transposable elements, plasmids | 0 | 0.0 | 0 | 0.0 | 0 | 0.0 |
| Membrane transport | 52 | 1.8 | 0 | 0.0 | 0 | 0.0 |
| Iron acquisition and metabolism | 11 | 0.4 | 0 | 0.0 | 0 | 0.0 |
| RNA metabolism | 45 | 1.6 | 0 | 0.0 | 0 | 0.0 |
| Nucleosides and nucleotides | 127 | 4.4 | 0 | 0.0 | 0 | 0.0 |
| Protein metabolism | 237 | 8.2 | 2 | 2.6 | 0 | 0.0 |
| Cell division and cell cycle | 0 | 0.0 | 0 | 0.0 | 0 | 0.0 |
| Motility and chemotaxis | 0 | 0.0 | 0 | 0.0 | 0 | 0.0 |
| Regulation and cell signaling | 32 | 1.1 | 4 | 5.2 | 0 | 0.0 |
| Secondary metabolism | 9 | 0.3 | 0 | 0.0 | 0 | 0.0 |
| DNA metabolism | 92 | 3.2 | 1 | 1.3 | 1 | 33.3 |
| Fatty acids, lipids, and isoprenoids | 286 | 9.9 | 7 | 9.1 | 0 | 0.0 |
| Nitrogen metabolism | 50 | 1.7 | 2 | 2.6 | 0 | 0.0 |
| Dormancy and sporulation | 3 | 0.1 | 0 | 0.0 | 0 | 0.0 |
| Respiration | 131 | 4.5 | 7 | 9.1 | 0 | 0.0 |
| Stress response | 61 | 2.1 | 0 | 0.0 | 2 | 66.7 |
| Metabolism of aromatic compounds | 139 | 4.8 | 2 | 2.6 | 0 | 0.0 |
| Amino acids and derivatives | 587 | 20.2 | 16 | 20.8 | 0 | 0.0 |
| Sulfur metabolism | 47 | 1.6 | 0 | 0.0 | 0 | 0.0 |
| Phosphorus metabolism | 36 | 1.2 | 0 | 0.0 | 0 | 0.0 |
| Carbohydrates | 514 | 17.7 | 25 | 32.5 | 0 | 0.0 |

Subsystem coverage – percentage of genes assigned to known biological functions

**Table S6**. Secondary metabolite clusters identified in *Rhodococcus opacus* 1CP with antiSMASH5.1.2.

| Region | | Type | From | To | Most similar known cluster | | Similarity |
| --- | --- | --- | --- | --- | --- | --- | --- |
| 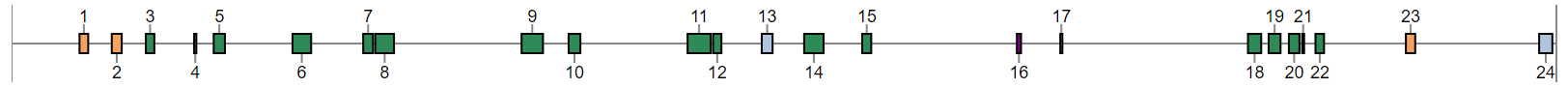 | | | | | | | |
| 1 | C | T1PKS | 338,090 | 381,328 | - |  | - |
| 2 | C | T1PKS | 498,582 | 548,204 | Tetrocarcin A | Polyketide | 11 % |
| 3 | C | NRPS-like | 668,194 | 710,420 | - |  | - |
| 4 | C | Butyrolactone | 908,453 | 919,379 | Herboxidiene | Polyketide | 3 % |
| 5 | C | NRPS | 1,005,799 | 1,061,615 | Erythrochelin | NRP | 57 % |
| 6 | C | NRPS | 1,398,057 | 1,490,744 | Rimosamide | NRP | 14 % |
| 7 | C | NRPS | 1,749,723 | 1,798,790 | - |  | - |
| 8 | C | NRPS | 1,809,674 | 1,903,199 | Mycinamicin II | Polyketide | 14 % |
| 9 | C | NRPS | 2,537,887 | 2,644,490 | Fengycin | NRP | 20 % |
| 10 | C | NRPS | 2,771,987 | 2,830,490 | Sch47554 / Sch47555 | Polyketide | 3 % |
| 11 | C | NRPS | 3,363,914 | 3,479,330 | Borrelidin | Polyketide | 6 % |
| 12 | C | NRPS-like | 3,490,749 | 3,532,283 | - | - | - |
| 13 | C | Terpene, T1PKS | 3,733,132 | 3,786,986 | 4-Hexadecanoyl-3-hydroxy-2-(hydroxymethyl)-2H-furan-5-one | Polyketide | 27 % |
| 14 | C | NRPS | 3,944,094 | 4,041,015 | Oxalomycin B | NRP + Polyketide | 12 % |
| 15 | C | NRPS | 4,232,947 | 4,278,020 | Phosphonoglycans | Saccharide | 5 % |
| 16 | C | Terpene | 5,002,595 | 5,023,536 | Carotenoid | Terpene | 18 % |
| 17 | C | Ectoine | 5,218,765 | 5,229,163 | Ectoine | Other | 75 % |
| 18 | C | NRPS | 6,152,660 | 6,218,679 | Chondrochloren A | NRP + Polyketide:Modular type I | 11 % |
| 19 | C | NRPS | 6,255,456 | 6,314,846 | Rhodochelin | NRP | 100 % |
| 20 | C | NRPS | 6,356,305 | 6,409,037 | Lobosamide A / Lobosamide B / Lobosamide C | Polyketide | 4 % |
| 21 | C | Bacteriocin | 6,422,585 | 6,432,143 | - | - | - |
| 22 | C | NRPS | 6,488,682 | 6,531,349 | - | - | - |
| 23 | C | hglE-KS | 6,938,594 | 6,984,980 | - | - | - |
| 24 | C | NRPS, Bacteriocin | 7,601,762 | 7,667,462 | - | - | - |
| 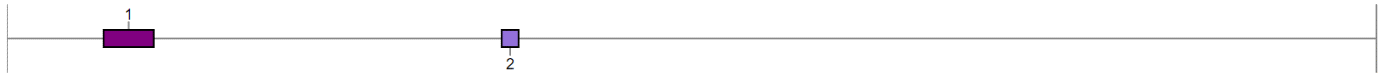 | | | | | | | |
| 1 | I | betalactone | 62,568 | 94,981 | - | - | - |
| 2 | I | butyrolactone | 319,897 | 330,823 | - | - | - |

Location of the putative secondary metabolite clusters are indicated by (C) chromosome and (Ι) pR1CP1. No secondary metabolite clusters were found on plasmid pR1CP2.

**Table S7**. Summary of upregulation of transcripts with T^WT^ as reference

|  | S^WT^ | P^WT^ | C^Δ123^ | C^Δ123^ | T^WT^ |
| --- | --- | --- | --- | --- | --- |
| Total mapped CDS | 7123 | 7766 | 7859 | 7862 | 7766 |
| Upregulated CDS | 631 | 1459 | 740 | 1776 | 1300 |
| Upregulated CDS (%) | 8 | 19 | 9 | 23 | 17 |

T – tetradecan, S – styrene, P – phenol, C – *p*-Cresol. A CDS is defined as upregulated if lfc > 0.58 and padj < 0.05.

**Table S8:** Specific activity of dioxygenase activity recorded for WT and ∆123 cell free crude extract on catechol(s)

| **Substrate/pathway** | **Ortho-cleavage**  **(specific activity mU/mg_crude_)** | | **Meta-cleavage**  **(specific activity mU/mg_crude_)** | |
| --- | --- | --- | --- | --- |
|  | **WT** | **∆123** | **WT** | **∆123** |
| Catechol | 98.1±0.6 | 9.2±1 | 0.2±0.3 | 1.2±0.005 |
| 4-methyl catechol | - | - | 0.2±0.3 | 3.4±0.2 |

**Table S9:** Values of Michaelis-Menten kinetics and turnover number k_cat_ of RoSOI1 and RoSOI2 for (R/S)-styrene oxide

| Enzyme | *K_M_* _(mM)_ | V_max_  _(µmoles_ _min_^-1^ _mg_^-1^_)_ | *k_cat_* | *k_cat_*/*K_M_* |
| --- | --- | --- | --- | --- |
| *Ro*SOI1 | 0.05±0.01 | 299±16 | 105±5 s^-1^ | 2.1x10^6^ s^-1^ M^-1^ |
| *Ro*SOI2 | 0.12±0.01 | 374±13 | 131±5 s^-1^ | 1.09x10^6^ s^-1^ M^-1^ |

**Table S10.** Plasmids used to construct knockout-mutant 1CP^Δ123^

| Plasmid | Inactivation of | Number of homologous nucleotides (left and right from inactivation insert) | Size of inactivation insert [nucleotides] |
| --- | --- | --- | --- |
| pK18A1-1EN1X4 | pheA1(1) / pROPH1 | 1225 / 464 | 344 (XhoI-fragment of pROPH8) |
| pK18A1-3E91 | pheA1(2) / pROPH3 | 1158 / 320 | 333 (EcoO109I -fragment of pROPH3) |
| pK18A1-8P1E2X1 | pheA1(3) / pROPH8 | 1415 / 211 | 344 (XhoI-fragment of pROPH8) |

**Table S11.** Strains and plasmids used in this study.

| **Strains and plasmids** | **Relevant properties** |  |
| --- | --- | --- |
| pMAL-c2X | Ap^r^; maltose-binding protein |  |
| pK18mobsacB | Km^r^; *oriT*, *sacB*, *lacZa* |  |
| pROPH1-1 | Ap^r^; 1.5 kb BamH I fragment of genomic DNA of *Rhodococcus opacus* 1CP in pBluescriptIIKS(+) |  |
| pROPH1-2 | Ap^r^; 3.7 kb EcoRI fragment of genomic DNA of *Rhodococcus opacus* 1CP in pBluescriptIIKS(+), overlap with pROPH1-1 |  |
| pROPH1-3 | Ap^r^; 5.0 kb NotI fragment of genomic DNA of *Rhodococcus opacus* 1CP in pBluescriptIIKS(+), overlap with pROPH1-1 |  |
| pROPH1-4 | Ap^r^; 4.2 kb EcoO109I fragment of genomic DNA of *Rhodococcus opacus* 1CP in pBluescriptIIKS(+), overlap with pROPH1-2 |  |
| pROPH3 | Ap^r^; 3.0 kb BamHI fragment of genomic DNA of *Rhodococcus opacus* 1CP in pBluescriptIIKS(+) |  |
| pROPH8 | Ap^r^; 8.0 kb BamH I fragment of genomic DNA of *Rhodococcus opacus* 1CP in pBluescriptIIKS(+) |  |
| pK18A1-1EN1X4 | Km^r^; pK18mobsacB carrying a 1.7 kb NotI/EcoRI-fragment of pROPH1-2 with a 330 bp insertion at the XhoI site |  |
| pK18A1-3E91 | Km^r^; pK18mobsacB carrying a 1.5 kb BamHI/ EcoRI-fragment of pROPH3 with a 333 bp insertion at the EcoO109I site |  |
| pK18A1-8P1E2X1 | Km^r^; pK18mobsacB carrying a 1.5 kb PstI/EcoRI-fragment of pROPH8 with a 344 bp insertion at the XhoI site |  |
| *Escherichia coli* DH5α | F^-^ φ80d*lacZ∆*M15 ∆(*lacZYA-argF*)U169 *endA*1 *recA*1 *hsdR*17 (r_K_^-^ m_K_^+^) *thi-*1 *supE*44 *λ*^-^ *gyrA*96 *relA*1 |  |
| *Escherichia coli* S17-1 | recA pro hsdR RP4­2­Tc::Mu­Km::Tn7 |  |
|  |  |  |

**Table S12.** Summary of transcriptome sequencing statistics of *Rhodococcus opacus* 1CP.

|  | Phenol^WT^ | | | | p-Cresol^Δ123^ | | | | Styrene^WT^ | | | Tetradecane^WT^ |
| --- | --- | --- | --- | --- | --- | --- | --- | --- | --- | --- | --- | --- |
|  | A | B | C | D | A | B | C | D | A | B | C | combined |
| Mappings | 5901083 | 5941975 | 3362212 | 7795041 | 5420259 | 11675713 | 8044628 | 9449227 | 7702164 | 8197683 | 7759711 | 672822 |
| Unique mapped reads | 5901083 | 5941975 | 3362212 | 7795041 | 5420259 | 11675713 | 8044628 | 9449227 | 7702164 | 8197683 | 7759711 | 672822 |
| Single Perfect Mappings | 5403436 | 5503018 | 3114270 | 7110072 | 4547166 | 10015747 | 6907121 | 7965936 | 7538501 | 7600536 | 7320376 | 641395 |
| Perfect Mappings | 0 | 0 | 0 | 0 | 0 | 0 | 0 | 0 | 0 | 0 | 0 | 0 |
| Single Best-Match Mappings | 497647 | 438957 | 247942 | 684969 | 773093 | 1659966 | 1137507 | 1483291 | 163663 | 597147 | 439335 | 31427 |
| Best-Match Mappings | 0 | 0 | 0 | 0 | 0 | 0 | 0 | 0 | 0 | 0 | 0 | 0 |
| Common Mappings | 0 | 0 | 0 | 0 | 0 | 0 | 0 | 0 | 0 | 0 | 0 | 0 |
| Single Perfect Coverage (%) | 73.25 | 72.85 | 67.30 | 75.06 | 75.53 | 80.55 | 78.13 | 81.20 | 44.32 | 66.05 | 65.12 | 69.67 |
| Perfect Coverage (%) | 0 | 0 | 0 | 0 | 0 | 0 | 0 | 0 | 0 | 0 | 0 | 0 |
| Single Best-Match Coverage (%) | 7.52 | 7.40 | 6.32 | 7.73 | 8.13 | 9.08 | 8.49 | 9.41 | 6.90 | 8 | 7.53 | 6.23 |
| Best-Match Coverage (%) | 0 | 0 | 0 | 0 | 0 | 0 | 0 | 0 | 0 | 0 | 0 | 0 |
| Common Match Coverage (%) | 0 | 0 | 0 | 0 | 0 | 0 | 0 | 0 | 0 | 0 | 0 | 0 |
| Total Coverage (%) | 80.77 | 80.26 | 73.62 | 82.79 | 83.66 | 89.63 | 86.62 | 90.61 | 51.22 | 74.05 | 72.65 | 75.90 |

**Table S13.** Annotation and transcriptome data of *R.opacus* 1CP *meta*-cleavage and side chain pathway in attacking *p*-cresol and styrene, respectively.

| Locus  [R1CP_RS] | Accession | Annotation | | Log_2_-fc | padj |
| --- | --- | --- | --- | --- | --- |
| **Transcriptome data of the meta-cleavage pathway (tetradecane as control vs *p*-cresol as treatment)** | | | | | |
| 00830 | WP_005260745 | C23DO | 4-Methylcatechol 23-dioxygenase | 11.7 | 1.7E-36 |
| 00835 | WP_011596410 | PD | Propanal dehydrogenase | 9 | 9.8E-21 |
| 00840 | WP_005260743 | HOHA | 4-Hydroxy-2-oxohexanoate aldolase | 9.3 | 8.0E-22 |
| 00845 | WP_005572674 | HMSD | 2-Methyl-2-hydroxymuconic semialdehyde dehydrogenase | 10.2 | 6.5E-28 |
| 00850 | WP_007296210 |  | SDR family oxidoreductase | 7.8 | 8.8E-15 |
| 00855 | WP_007296209 | HMAT | 5-Methyl-2-hydroxymuconate tautomerase | 7.7 | 8.6E-10 |
| 00860 | WP_005572670 | NphA2 | 4-Nitrophenol 2-monooxygenase reductase | 10.8 | 5.2E-45 |
| 00865 | WP_065488481 | NphA1 | 4-Nitrophenol 2-monooxygenase | 13.4 | 3.2E-42 |
| 00870 | WP_065492837 | NphR | NphR transcriptional regulator | 5.9 | 8.8E-18 |
| 00875 | WP_005260736 |  | EthD family reductase | 7.7 | 1E-09 |
| 00880 | WP_065488482 | GcoB | Propable aromatic O-demethylase reductase subunit | 5.8 | 6.6E-17 |
| 00885 | WP_065488484 | GcoA | Propable aromatic O-demethylase cytochrome P450 subunit | 7.9 | 1.7E-21 |
| 00890 | WP_065488486 | AraC | AraC family transcriptional regulator | 5.7 | 9.5E-10 |
| 00895 | WP_065488488 | PCoAD | Propanoyl-CoA-dehydrogenase | 9.2 | 7.8E-29 |
| 00900 | WP_065488490 | HMAT | 5-Methyl-2-hydroxymuconate tautomerase | 9.3 | 3.8E-16 |
| 00905 | WP_065488493 | OMMC | 2-Oxo-5-methyl-cis-muconate decarboxylase | 10.7 | 1.6E-23 |
| 00910 | WP_005260729 | HHDH | 2-Hydroxyhexa-24-dienoate hydratase | 10.8 | 2.7E-24 |
| 00915 | WP_043793345 | IclR | IclR family transcriptional regulator | 9.6 | 1E-31 |
| 02725 | WP_081315251 | HMSH | 2-Hydroxymuconate semialdehyde hydrolase | 0.08 | 1.0 |
| 12650 | WP_005563278 | PCoAC | Propanoyl-CoA-carboxylase subunit epsilon | 1.5 | 0.02 |
| 12655 | WP_065490413 | PCoAC | Propanoyl-CoA-carboxylase subunit beta | 1.3 | 0.02 |
| 20790 | WP_065491510 | HOHA | 4-Hydroxy-2-oxohexanoate aldolase | 6.9 | 1.2E-7 |
| 20795 | WP_065493144 | PD | Propanal dehydrogenase | 5.5 | 1.9E-4 |
| 20800 | WP_005260580 | HHDH | 2-Hydroxyhexa-24-dienoate hydratase | 4.8 | 4.0E-5 |
| 22245 | WP_065491632 |  | 45-DOPA dioxygenase extradiol | 4.6 | 3E-12 |
| 23270 | WP_005261204 | PCoAC | Propanoyl-CoA-carboxylase subunit beta | 1.4 | 0.02 |
| **Transcriptome data of upregulated genes involved in styrene degradation (*p*-cresol as control vs styrene as treatment)** | | | | | |
| 30630 | WP_005250812 |  | hypothetical protein | 11 | 3.7E-59 |
| 30635 | WP_005255970 | FixL | PAS domain-containing sensor histidine kinase | 7.6 | 2.6E-82 |
| 30640 | WP_005255969 | HsaA | Flavin-dependent monooxygenase oxygenase subunit | 11 | 1.6E-59 |
| 30645 | WP_005255968 |  | hypothetical protein | 11.3 | 6.7E-48 |
| 30650 | WP_005255967 |  | nuclear transport factor 2 family protein | 11.0 | 1.8E-59 |
| 30655 | WP_005574578 | AntA | Anthranilate 12-dioxygenase large subunit (alpha) | 12.7 | 4.0E-61 |
| 30660 | WP_005255965 | BphE | Biphenyl dioxygenase subunit beta | 11.7 | 1.9E-40 |
| 30665 | WP_005255964 | FabG | SDR family oxidoreductase | 10.5 | 1.3E-44 |
| 30670 | WP_005255963 | NdmD | oxidoreductase | 11 | 4.7E-72 |
| 30675 | WP_005250795 | TmoT | response regulator transcription factor (toluene degradation) | 8.4 | 1.2E-88 |
| 30680 | WP_065492337 | HpaC | 4-hydroxyphenylacetate 3-monooxygenase reductase subunit | 12.5 | 3.7E-44 |
| 30685 | WP_065492338 | YwcB | DUF485 domain-containing protein | 11.8 | 6.2E-30 |
| 30690 | WP_065492339 | YwcA | cation acetate symporter | 11 | 2.7E-41 |
| 30695 | WP_065492340 |  | amidase | 6.8 | 1.4E-08 |
| 30705 | WP_065492342 |  | Aryl acylamidase | 4.9 | 1.3E-21 |
| 30710 | WP_061046101 | AraC | helix-turn-helix domain-containing protein | 3.9 | 2.0E-17 |
| 30715 | WP_054245641 | PaaK | Phenylacetate-coenzyme A ligase | 6.8 | 8.2E-65 |
| 30720 | WP_054245642 | PaaE | 12-phenylacetyl-CoA epoxidase subunit E | 7.4 | 1.5E-49 |
| 30725 | WP_005566102 | PaaD | 12-phenylacetyl-CoA epoxidase subunit D | 8 | 5.5E-50 |
| 30730 | WP_065493296 | PaaC | 12-phenylacetyl-CoA epoxidase subunit C | 6.8 | 1.3E-69 |
| 30735 | WP_009475635 | PaaB | 12-phenylacetyl-CoA epoxidase subunit B | 8.3 | 1E-49 |
| 30740 | WP_065492343 | PaaA | 12-phenylacetyl-CoA epoxidase subunit A | 7.5 | 7.3E-60 |
| 30745 | WP_065492344 | PaaG | 12-epoxyphenylacetyl-CoA isomerase | 3.8 | 5E-12 |
| 30750 | WP_065492345 | PaaH | 3-hydroxyadipyl-CoA dehydrogenase | 5.0 | 2.6E-16 |
| 30755 | WP_065492346 | PaaF | 23-dehydroadipyl-CoA hydratase | 4.7 | 1.9E-11 |
| 30760 | WP_009475639 | PaaJ | 3-oxoadipyl-CoA/3-oxo-56-dehydrosuberyl-CoA thiolase | 2.7 | 5.9E-06 |
| 30765 | WP_065492347 | PaaZ | bifunctional aldehyde dehydrogenase/enoyl-CoA hydratase | 5.3 | 2.8E-20 |
| 30775 | WP_005250762 | PaaT | Acyl-coenzyme A thioesterase PaaI | 4.9 | 8.9E-15 |
| 37785 | WP_081315606 | MetF | 510-methylenetetrahydrofolate reductase | 8 | 5.4E-46 |
| 37790 | WP_065493722 | LigM | Vanillate/3-O-methylgallate O-demethylase | 7.2 | 1.6E-58 |
| 37795 | WP_065493899 | PurU | Formyltetrahydrofolate deformylase | 6.7 | 2.2E-52 |
| 40665 | WP_081315607 | FolP1 | Dihydropteroate synthase | 4.4 | 6.9E-24 |
| 37805 | WP_065493900 | StyC | StyC-Styrene oxide isomerase | 7.6 | 1.2E-74 |
| 37810 | WP_081315654 | NoS | Nitric oxide synthase endothelial | 7.4 | 2E-34 |
| 37860 | WP_065493734 | NodI | Nod factor export ATP-binding protein I | 12.2 | 1E-54 |
| 37865 | WP_065493902 | StyD | StyD-Phenylacetaldehyde dehydrogenase | 11.9 | 1.8E-58 |
| 37870 | WP_065493735 | StyC | StyC-Styrene oxide isomerase | 13.0 | 1.7E-78 |
| 37875 | WP_065493736 | StyB | StyB-SMO flavin oxidoreductase | 12.9 | 2.0E-65 |
| 37880 | WP_065493737 | StyA | StyA-SMO styrene monooxygenase | 13.8 | 4.6E-81 |
| 37885 | WP_081315608 | AndR | Anthranilate 12-dioxygenase regulatory protein | 7 | 4.0E-68 |
| 37890 | WP_081315609 | NphR | Transcriptional activator NphR | 10 | 4.2E-75 |

**Table S14.** Comparative transcriptome data of PD630 grown on phenol vs 1CP^∆123^ grown on *p*-cresol. Upregulated and downregulated genes are marked in green and red, respectively, while the grey highlight indicates no significant changes in gene regulation.

| **Annotation** | **Gene ID [1CP]** | **Gene ID**  **[PD630]** |
| --- | --- | --- |
| Probable succinyl-CoA:3-ketoacid coenzyme A transferase subunit A | R1CP_00985  R1CP_24505 | LPD05449 |
| protocatechuate 34-dioxygenase beta subunit | R1CP_24510 | LPD05450 |
| protocatechuate 34-dioxygenase alpha subunit | R1CP_24515 | LPD05451 |
| 3-carboxy-ciscis-muconate cycloisomerase | R1CP_24520 | LPD05452 |
| 3-oxoadipate enol-lactonase | R1CP_30450 | LPD05453 LPD06743 |
| transcriptional regulator PcaR | R1CP_24530 | LPD05454 |
| Acetyl-CoA acetyltransferase | R1CP_00975  R1CP_04805 R1CP_04830 R1CP_07345 R1CP_07615 R1CP_09820 R1CP_24535 R1CP_25115 R1CP_25965 R1CP_30440 R1CP_32160 R1CP_32170 R1CP_34365 | LPD05455 |
| xylose repressor | R1CP_29605 | LPD06565 |
| muconolactone delta-isomerase | R1CP_29610 | LPD06566 |
| Muconate cycloisomerase 1 | R1CP_29615 R1CP_32000 | LPD06567 |
| Catechol 12-dioxygenase | R1CP_01035  R1CP_29620 R1CP_30430 | LPD06568 LPD06570 LPD06742 |
| phenol hydroxylase reductase component | R1CP_29650 R1CP_30420  R1CP_39645 | LPD06575 LPD06740 |
| phenol hydroxylase | R1CP_39650 R1CP_30420 | LPD06576 LPD06741 |
| Beta-ketoadipyl-CoA thiolase | R1CP_10490 R1CP_31465 R1CP_34655 R1CP_39975 | LPD06744 |
| shikimate transport protein | R1CP_31590 | LPD06699 LPD07505 |
| NDMA-dependent methanol dehydrogenase | R1CP_11675 | LPD02697 |
| 60 kDa chaperonin 3 | R1CP_19770 | LPD04557 |
| methane monooxygenase | R1CP_19735 | LPD04550 LPD04551 LPD04552 |

**
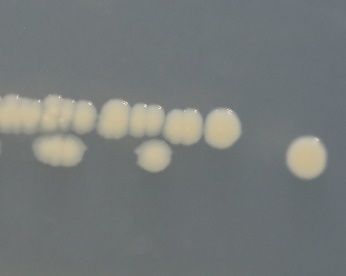
**

**Figure S1** Colonies of *Rhodococcus opacus* 1CP grown on solid MM.


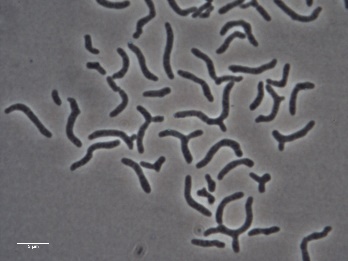


**Figure S2** Light microscopic image of 1CP during the exponential phase.


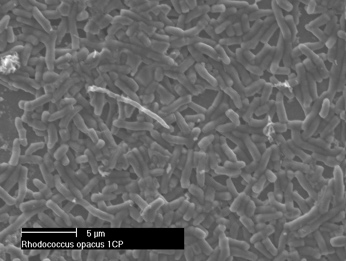

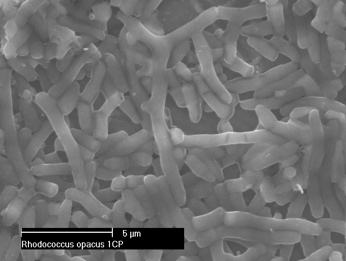




**Figure S3** Electron microscopy images of *Rhodococcus opacus* 1CP.


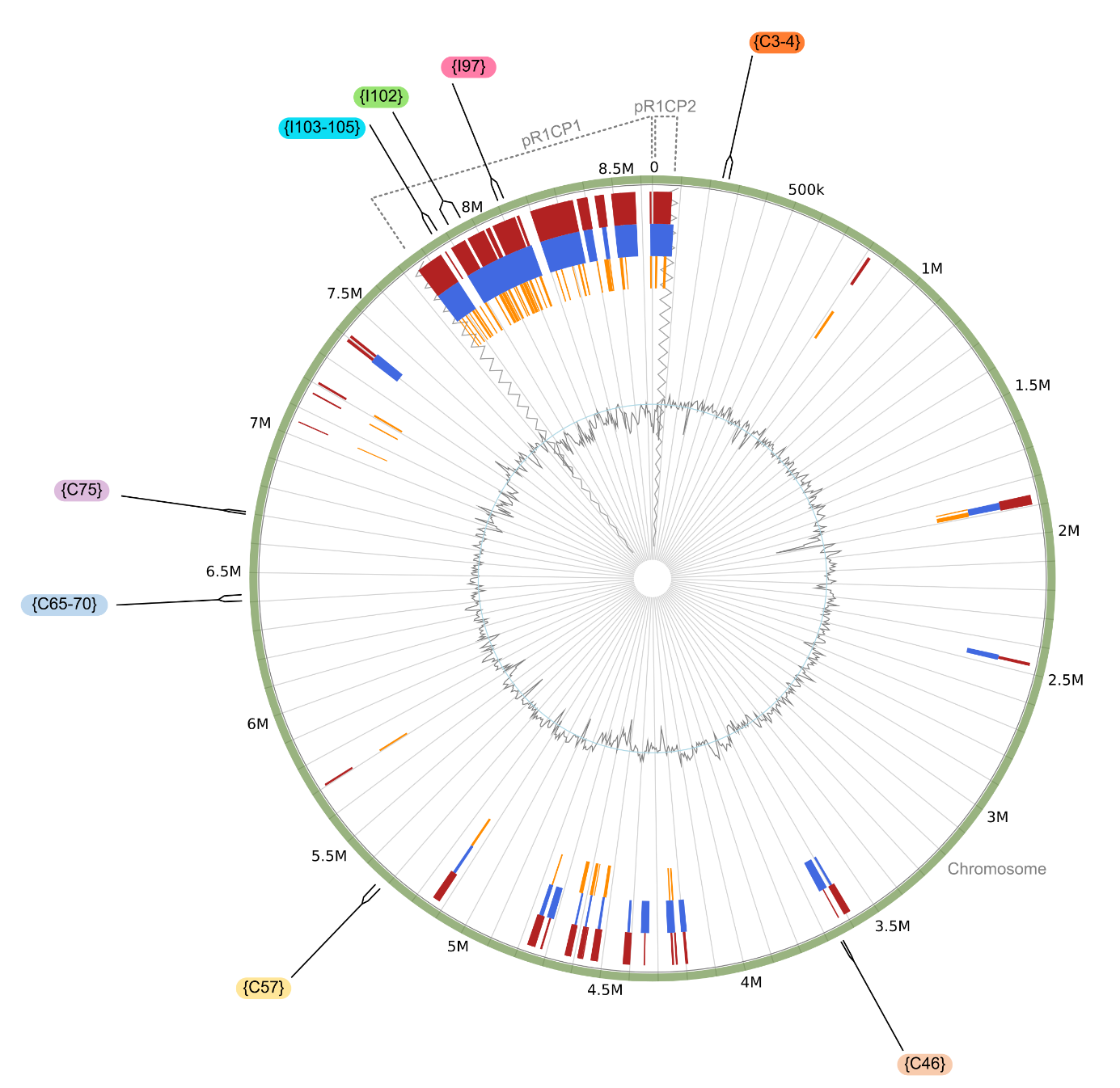


**Figure S4.** Horizontal gene transfer analysis of the complete genome of *Rhodococcus opacus* 1CP versus the reference genome of *Rhodococcus opacus* B4 using the software Islandviewer 4 (87). Regions representing the plasmids are depicted with grey dashed lines, while the remaining segments represents the chromosome. Genomic island predictions are shown in the circle as colored blocks (SIGI-HMM in orange, IslandPath-DIMOB in blue and integrated results in red). Relevant gene clusters of this study are indicated at the respective position on the genome following the same color code as found in table 1. The GC-content is visualized in the center of the genome blot.


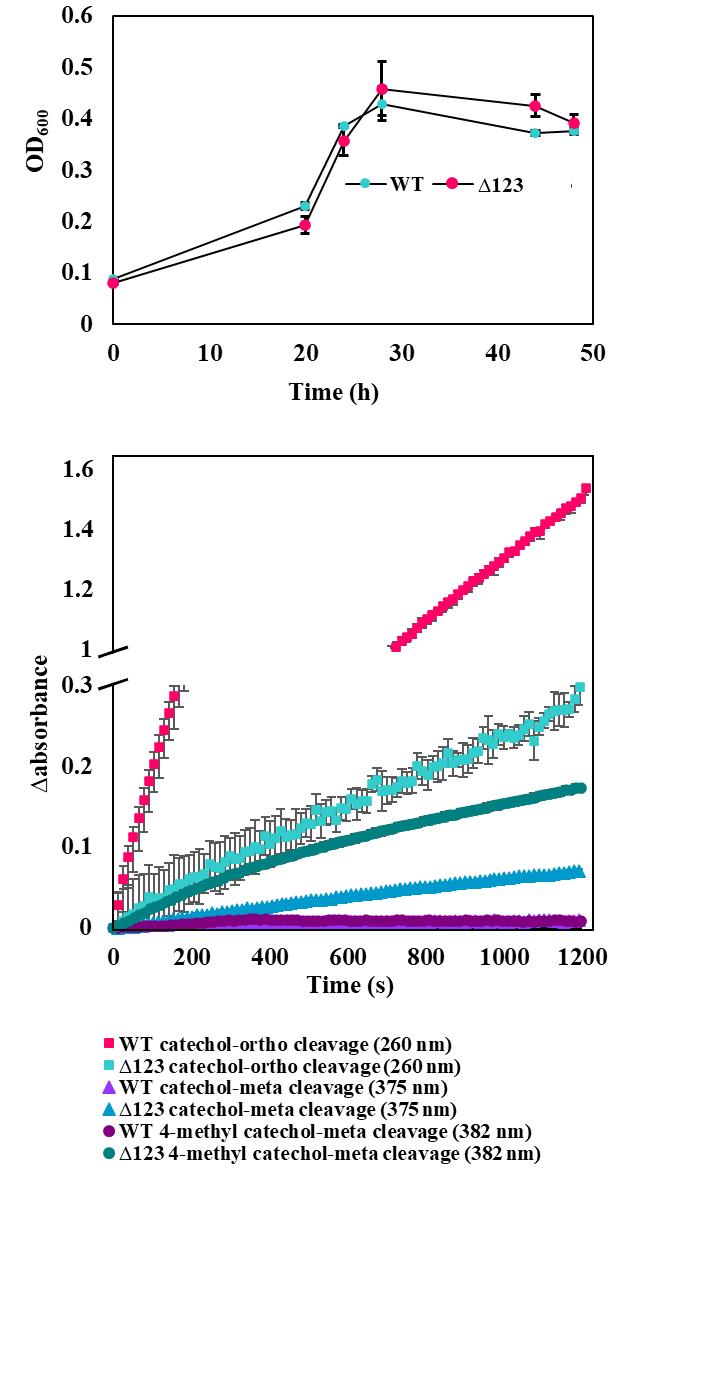


**Figure S5.** The growth curve (top graph) of WT and ∆123 when *p*-cresol is supplemented as a sole carbon source. The dioxygenase activity (catechol 1,2- and catechol 2,3-dioxygenase) of *p*-cresol grown WT and ∆123 tested on different substrates. The observed change in absorbance over time is given in y- and x-axis, respectively.


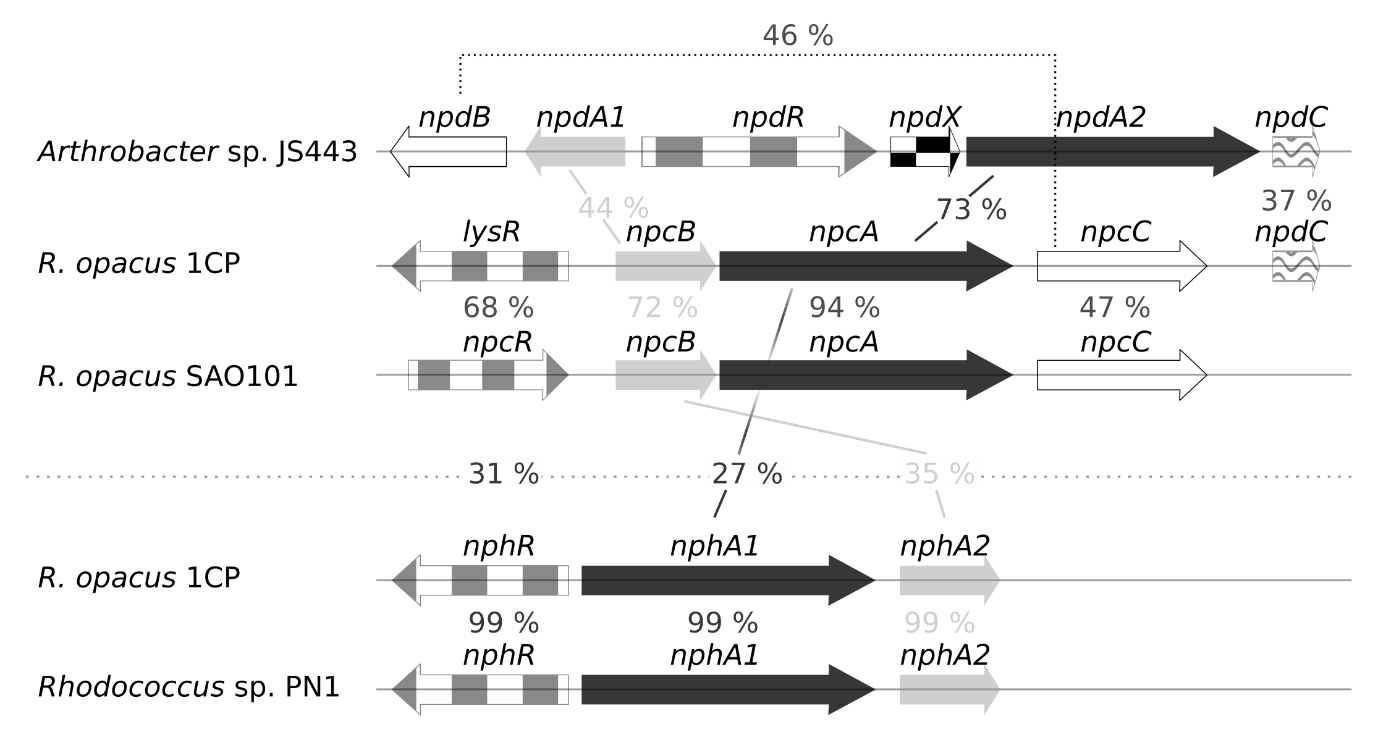


**Figure S6.** Comparison of degradation clusters initiating the degradation of *para*-substituted phenols in known rhodococci and *Arthrobacter.* The respective gene clusters can be reviewed on NCBI and the respective publication: *Arthrobacter sp.* JS443 - EF052871 (99), *R. opacus* SAO010 – AB154422 (97) *Rhodococcus* sp. PN1 – AB081773 (92) and on the genome of *R. opacus* 1CP. All percentage values report the sequence identity on the protein level towards the respective homolog in *R. opacus* 1CP.
